# Supplementary material for: Identification of Ephrin type-B receptor 4 as a critical mediator of tissue fibrosis
Source: JCI Insight. 2025 Dec 22;10(24):e189156. doi: 10.1172/jci.insight.189156 (PMC12890498; doi:10.1172/jci.insight.189156)
Supplement: Supplemental data [file jciinsight-10-189156-s125.pdf]

SUPPLEMENTAL MATERIAL

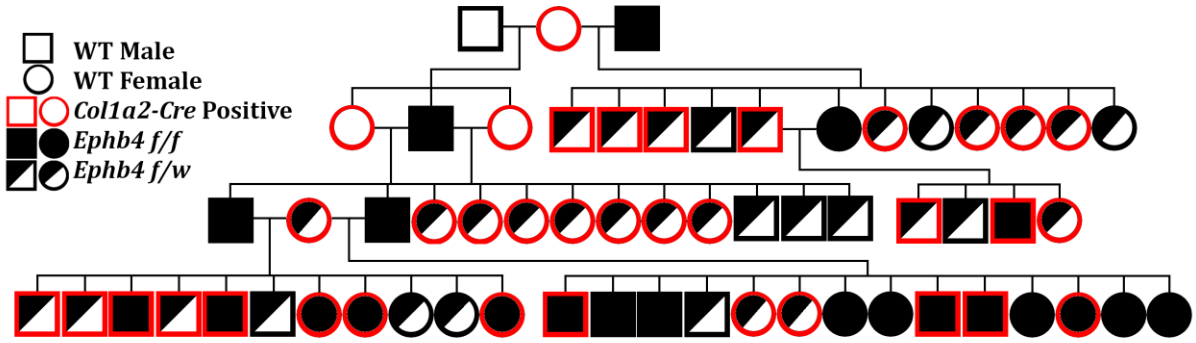

Supplementary Figure 1. Pedigree for the generation of *Col1a2*-driven *Ephb4* CKO mouse lines. *Ephb4*$^{tm1.1Jwu}$ mice were crossed with B6.Cg-Tg(*Col1a2*-Cre/ERT,-ALPP)7Cpd mice to generate a *Ephb4* conditional knockout (CKO) mouse line when treated with 4-hydroxytamoxifen at 4-5 weeks of age. Mice of F5 and greater were used for experiments in this study.

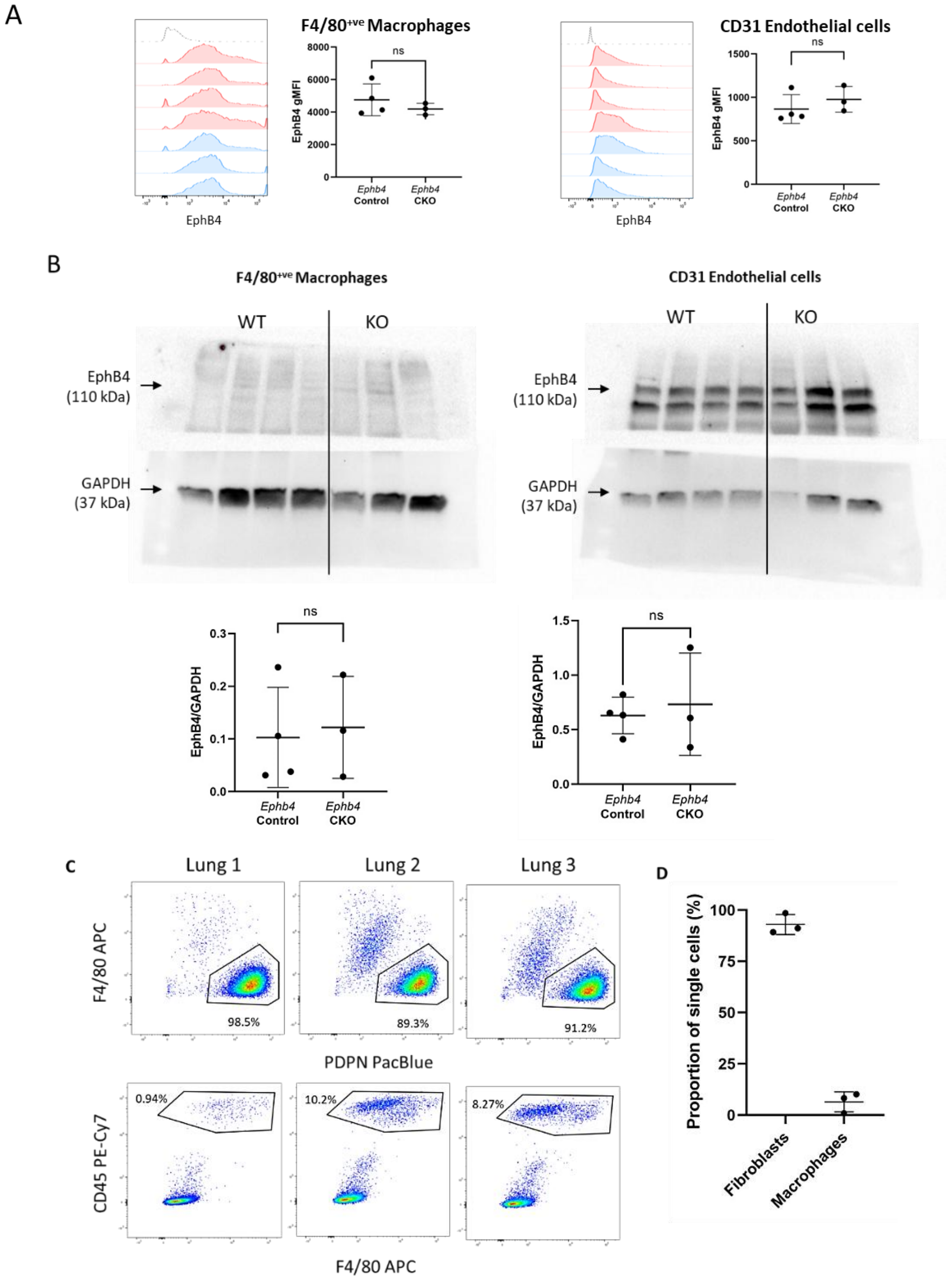

Supplementary Figure 2: EphB4 expression is not reduced in macrophages and endothelial cells of *Ephb4* conditional knockout (CKO) mice. Lungs were recovered from *Ephb4* control (n=4) and *Ephb4* CKO (n=3) mice and digested with collagenase to produce a single cell suspension. (A) *Ephb4* control (n=4) and *Ephb4* CKO (n=3) cells were labeled with anti-EphB4, F4/80, CD31 and a live/dead marker (eFluor780), and analyzed by flow cytometry. Relative surface expression of EphB4 was determined based on geometric mean fluorescence intensity (gMFI) after gating on macrophage and endothelial cell populations. Histograms of EphB4 expression (FITC channel) are shown including: unstained controls (dotted line), *Ephb4* control (red) and *Ephb4* CKO (blue). Data are expressed as mean ± SD where differences (ns, p>0.05) were identified using unpaired 2-tailed student's T test. (B) Macrophages (F4/80+ve) and endothelial (CD31+ve) cells were sorted on a BD FACS ARIA III and lysed in RIPA buffer. The equivalent of 120,000 sorted macrophages and 240,000 sorted endothelial cells were loaded per lane. EphB4 expression was determined by densitometry and mean expression relative to GAPDH is shown. Data are expressed as mean ± SD where differences (ns, p>0.05) were identified using unpaired 2-tailed student's T test. (C) Mouse

lungs were digested and recovered cells were cultured in standard culture media, passaged once, and grown to confluence. Cells were processed for flow cytometry using standard procedures. Scatterplots are shown indicating percentages of PDPN+ve and F4/80+ve populations for 3 independent mice. (D) Mean percentages (±StDev) of gated populations are shown for PDPN+ve fibroblasts and F40/80+ve, CD45+ve macrophages (n=3).

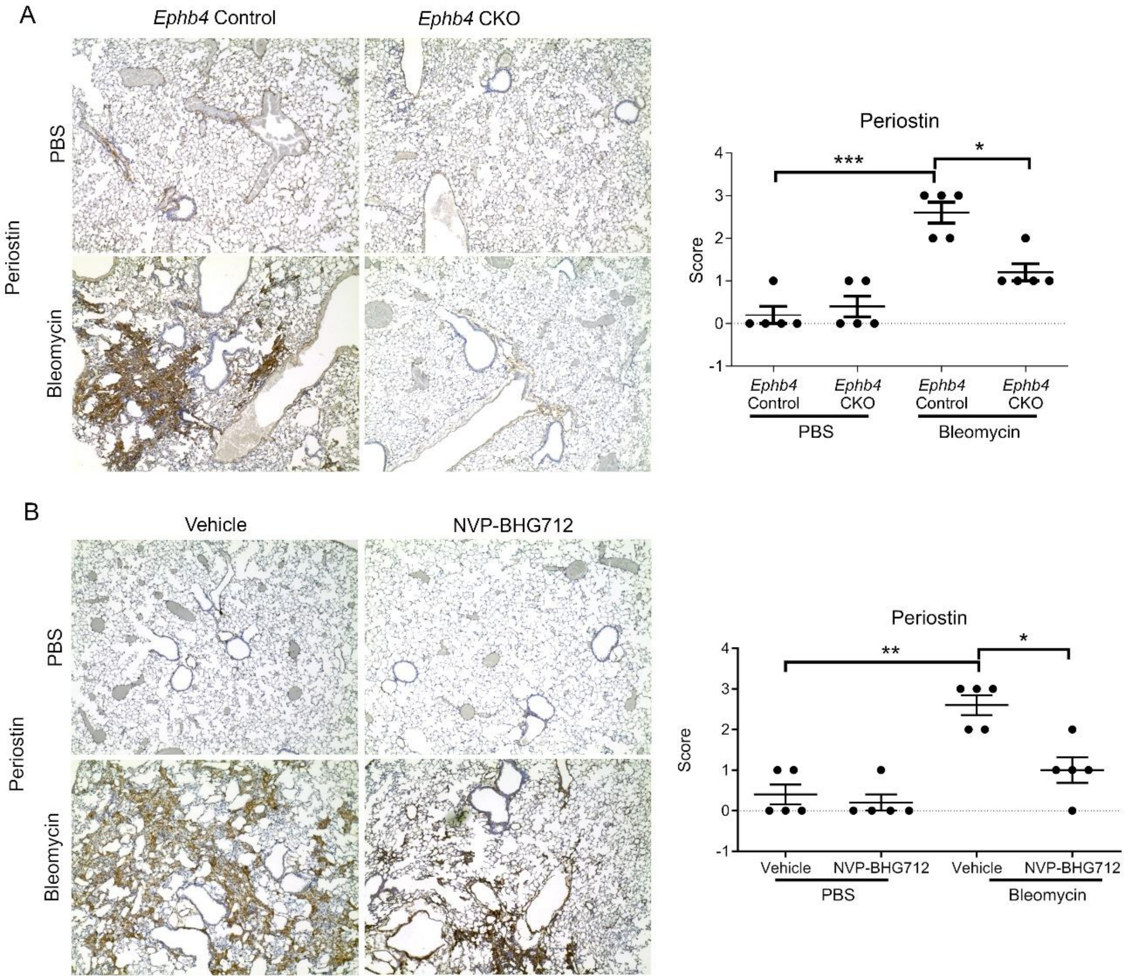

Supplementary Figure 3: EphB4 conditional knockout or inhibition by NVP-BHG712 reduces the expression of periostin in mouse lung tissue. PBS or bleomycin intratracheal instillation were performed on (A) *Ephb4* control and *Ephb4* CKO mice, or (B) mice treated with EphB4 inhibitor NVP-BHG712 or vehicle (n=6/group). Periostin staining was performed in lung tissue sections to assess myofibroblast populations in mouse lungs. Semi-quantitative scoring (0-3) of the staining intensity was assigned to each mouse lung. For all graphs, data are expressed as mean ± SD where significant differences (*, $p < 0.05$; **, $p < 0.01$; ***, $p < 0.001$) were identified using 2-way ANOVA with Tukey's post-hoc tests.

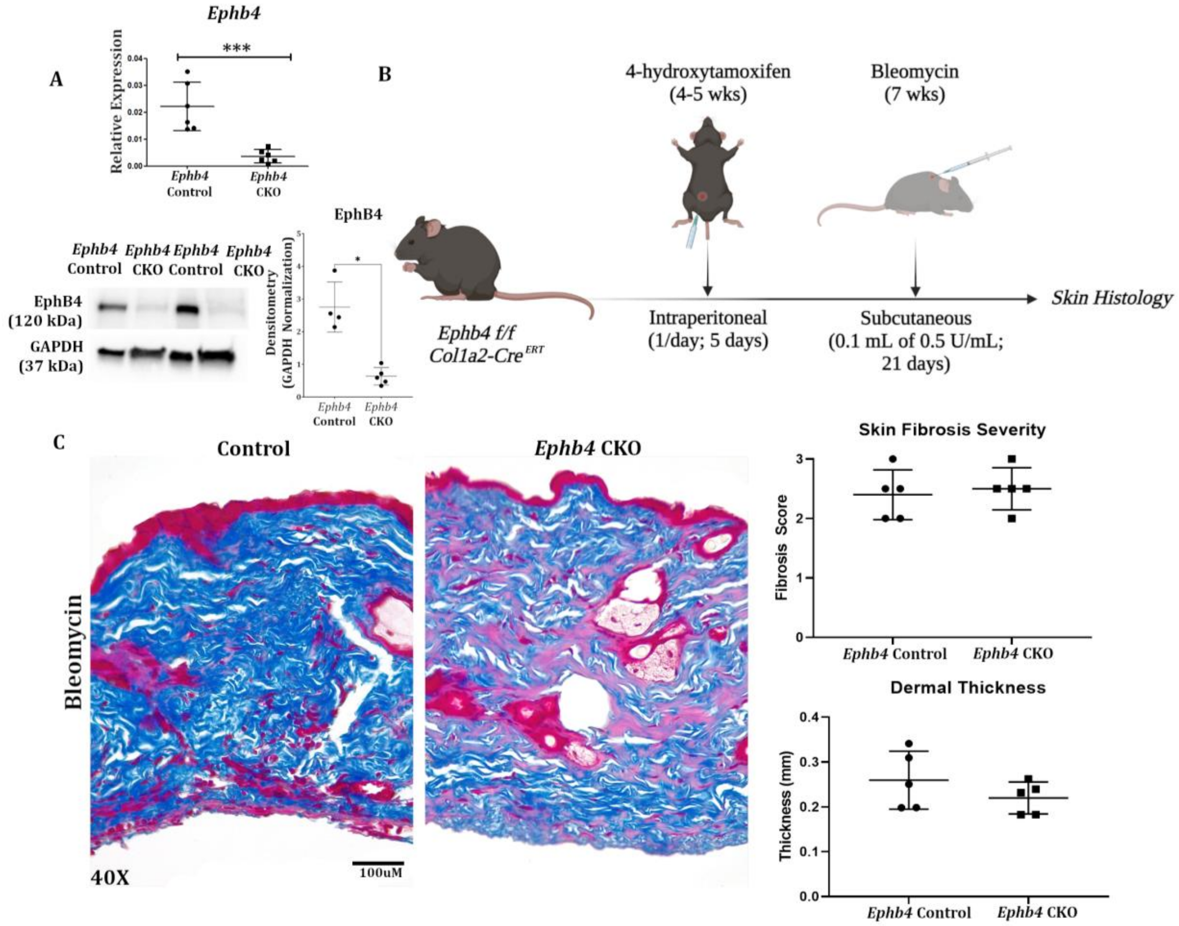

Supplementary Figure 4. Conditional knockdown of *Ephb4* in mouse fibroblasts does not confer protection in a skin fibrosis model. (A) Effective knockdown of *Ephb4* expression was observed at both the transcript (qRT-PCR, n=6) and protein levels (Western blotting; n=4, *Ephb4* control; n=5, *Ephb4* CKO) in P1 mouse skin fibroblasts isolated 7 days after final treatment with 4-hydroxytamoxifen (1 mg/day for 5 days; *i.p.*). Data is expressed as mean ± SD and significance was determined using unpaired 2-tailed student's T tests. *, $p<0.05$; ***, $p<0.001$. (B) *Ephb4$^{f/f}$;Col1a2-Cre^{ERT}* mice were treated with either 4-hydroxytamoxifen (*Ephb4* CKO) or corn oil (*Ephb4* control) at 4–5 weeks of age. To induce skin fibrosis, mice received subcutaneous injections of bleomycin (100 µL of 0.5 U/mL dissolved in PBS for 21 days). (C) Representative images of dermis sections stained with Gomori's One Step Trichrome from *Ephb4* CKO and *Ephb4* control mice (n=5, 4 sections/animal). To assess skin fibrosis severity, skin fibrosis scoring (0-3) and dermal thickness were quantified with no significant differences between groups found ($p< 0.05$). Data is expressed as mean ± SD and significance determined using unpaired 2-tailed student's T tests. ns, $p>0.05$.

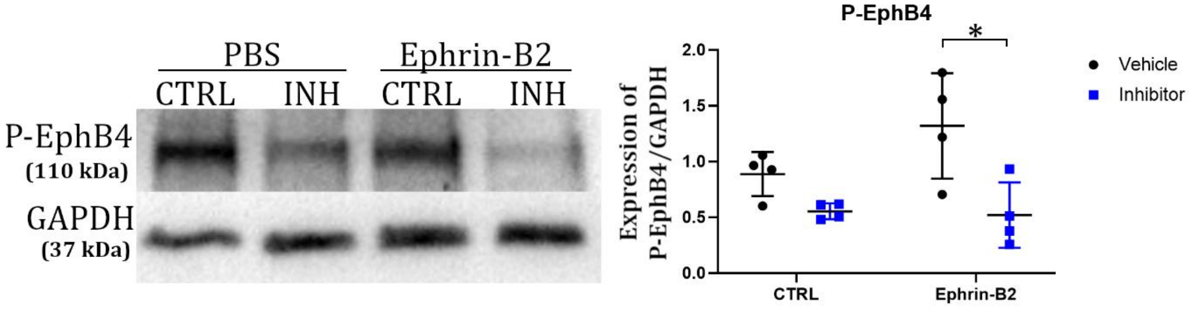

Supplementary Figure 5. NVP-BHG712 treatment of human lung fibroblasts inhibits EphB4 phosphorylation. Primary human lung fibroblasts were treated with pre-clustered Ephrin-B2 (1 µg/mL; or PBS control) and NVP-BHG712 (250 nM; or DMSO vehicle) for 24h (n=4). Western blotting was performed on protein lysates to detect phospho(P)-EphB4. GAPDH was used as a housekeeping control. Data is expressed as mean ± SD and significance was determined by 2-way ANOVA followed by Tukey's post-hoc tests adjusted for multiple comparisons. *, $p < 0.05$.

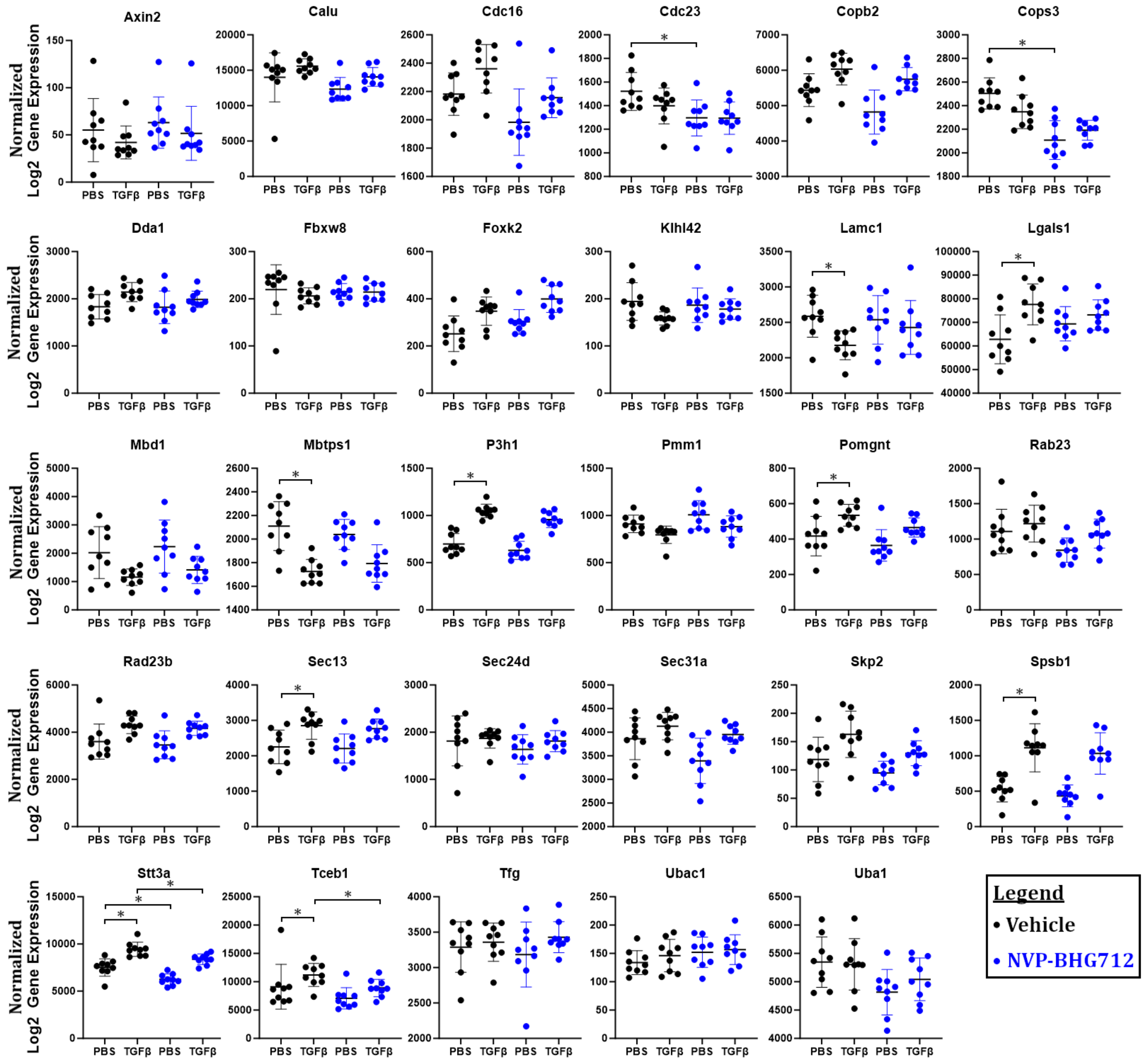

Supplementary Figure 6. Expression of remaining genes in the NanoString codeset. Transcript levels of the remaining genes not included in **Figure 4** in response to 24 h co-treatment of P4 mouse lung fibroblasts (n=9) with TGF-β1 (20 ng/mL; or PBS control) and NVP-BHG712 (250 nM; or DMSO vehicle). RNA was isolated and NanoString was performed to examine the transcript levels of downregulated genes identified by RNAseq that were linked to significantly enriched pathways. Data is expressed as mean ± SD. Wilcoxon signed rank test and FDR q≤0.05 performed to identify significant differences in gene expression between treatment groups. *, q < 0.05.

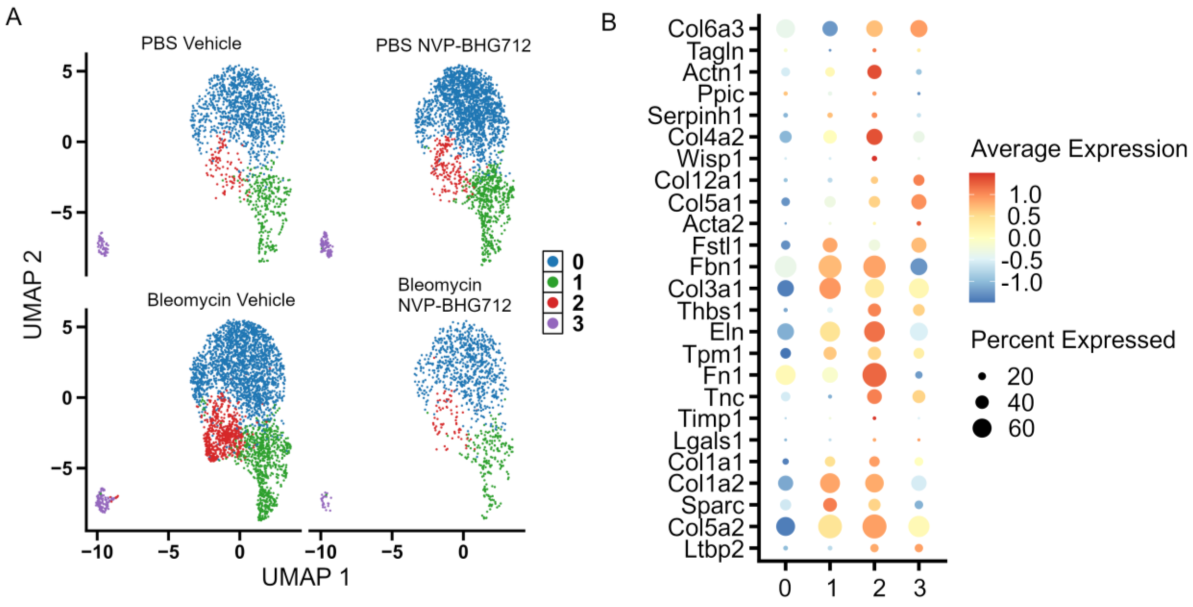

Supplementary Figure 7. Identification of fibroblast subclusters in mouse lungs and association of fibroblast subclusters with an activated fibroblast gene profile. (A) UMAP projection of distinct fibroblast populations identified in lung tissue from bleomycin- or PBS-treated mice that were administered NVP-BHG712 or vehicle. (B) Expression of key marker genes associated with activated fibroblasts from Peyser et al.[23]. were mapped to our snRNAseq dataset.

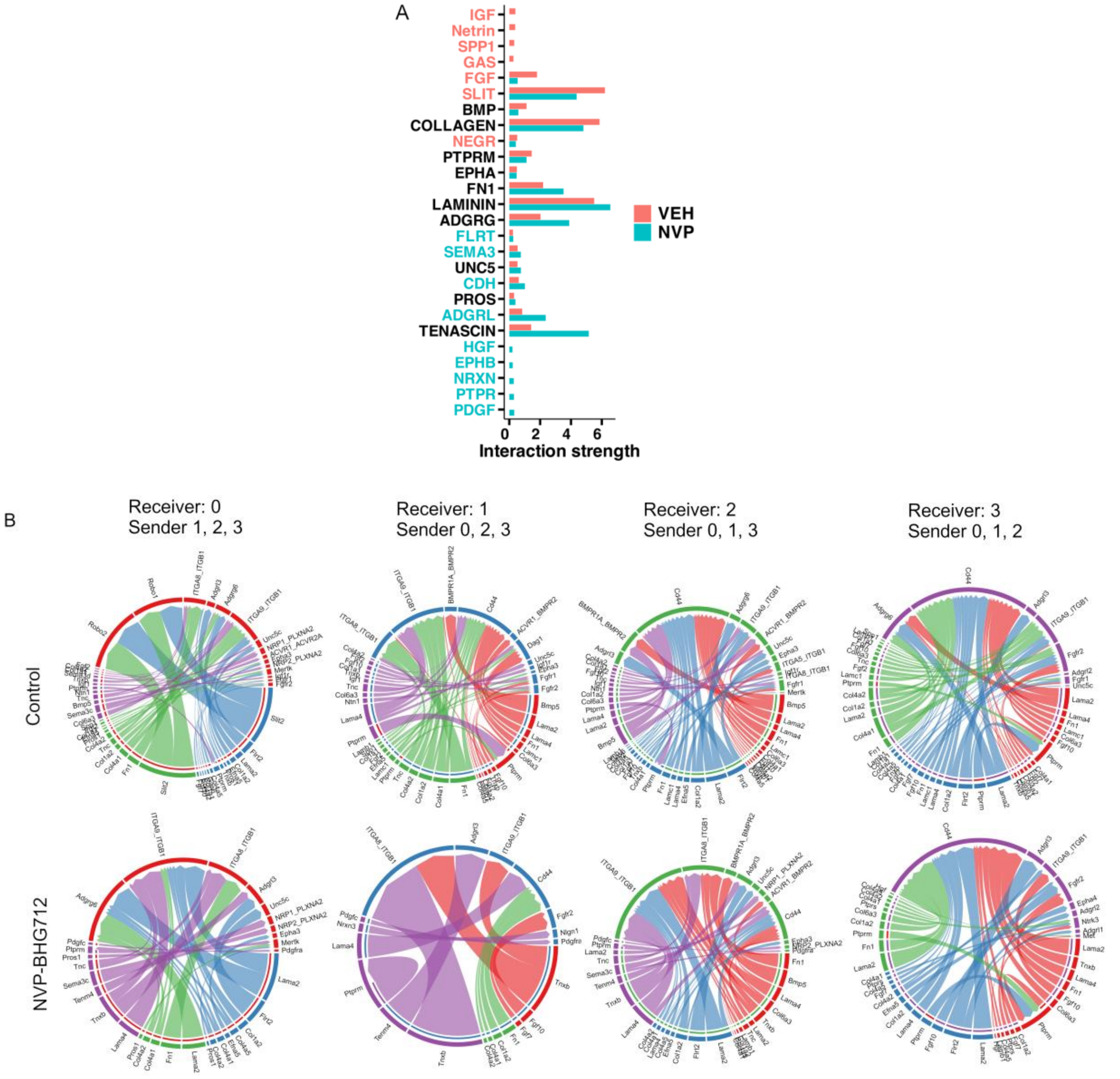

Supplementary Figure 8. Comparison of cell-cell interactions in lung fibroblasts from bleomycin-treated treated with vehicle compared to EphB4 inhibitor. (A) Cell-cell communication between fibroblast subclusters in bleomycin-treated vehicle and NVP-BHG712 groups showing significant interactions across various signaling pathways. (B) Comparison of the ligand-receptor pair interactions between vehicle and NVP-BHG712 that contribute to signaling between fibroblast subclusters.

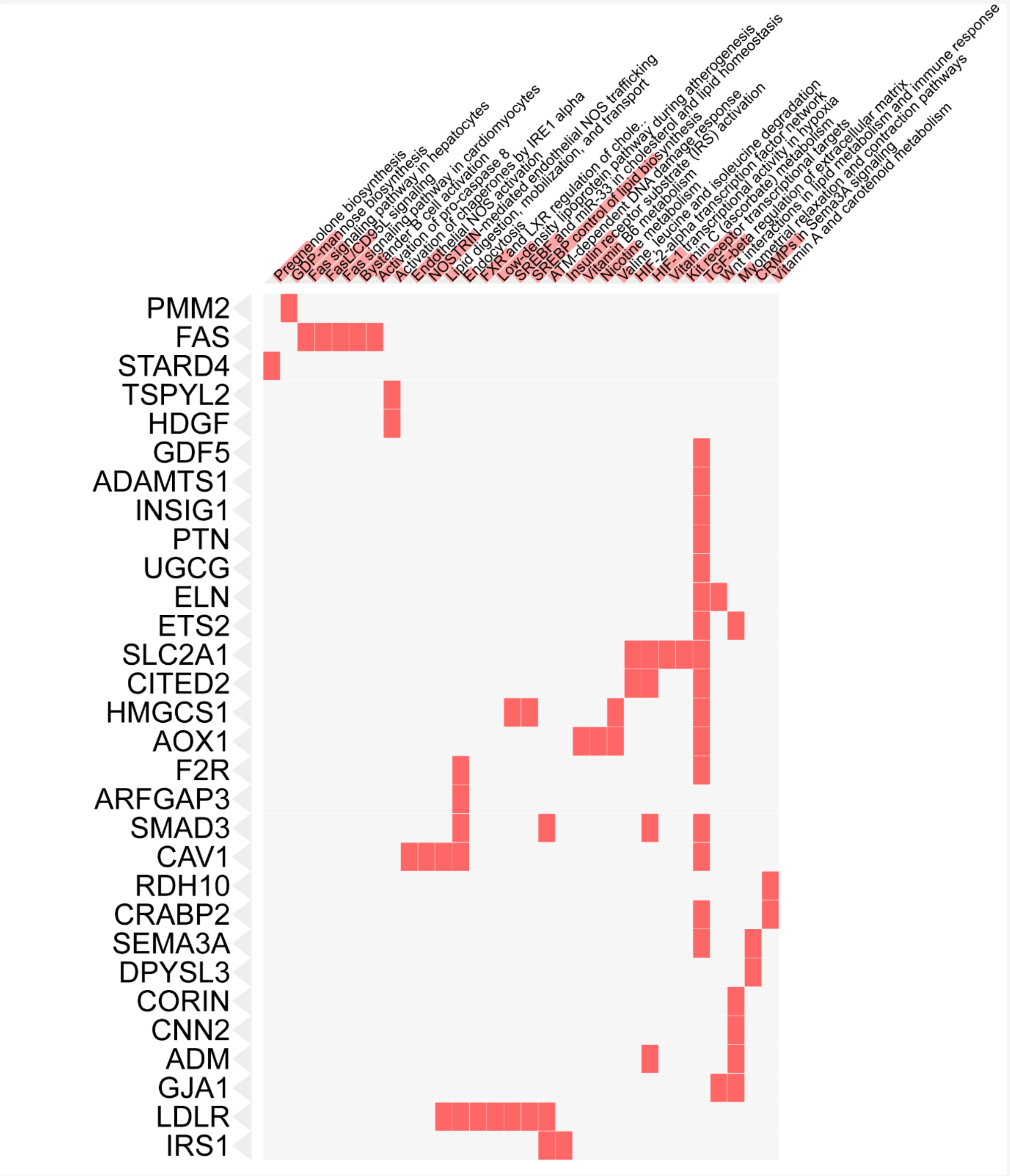

Supplementary Figure 9. GO enrichment analysis of transcriptomes of IPF fibroblasts treated with NVP-BHG712 compared to vehicle controls. Primary IPF patient fibroblasts were treated with NVP-BHG712 (250 nM) for 24 h and RNA was harvested for total RNA sequencing. DEGs were identified and pathway enrichment analysis was performed considering down regulated genes, using Enrichr web portal (June 1, 2022) and BioPlanet 2019 pathway sources. Highlighted association of input genes with enriched pathways is presented using combined scores.

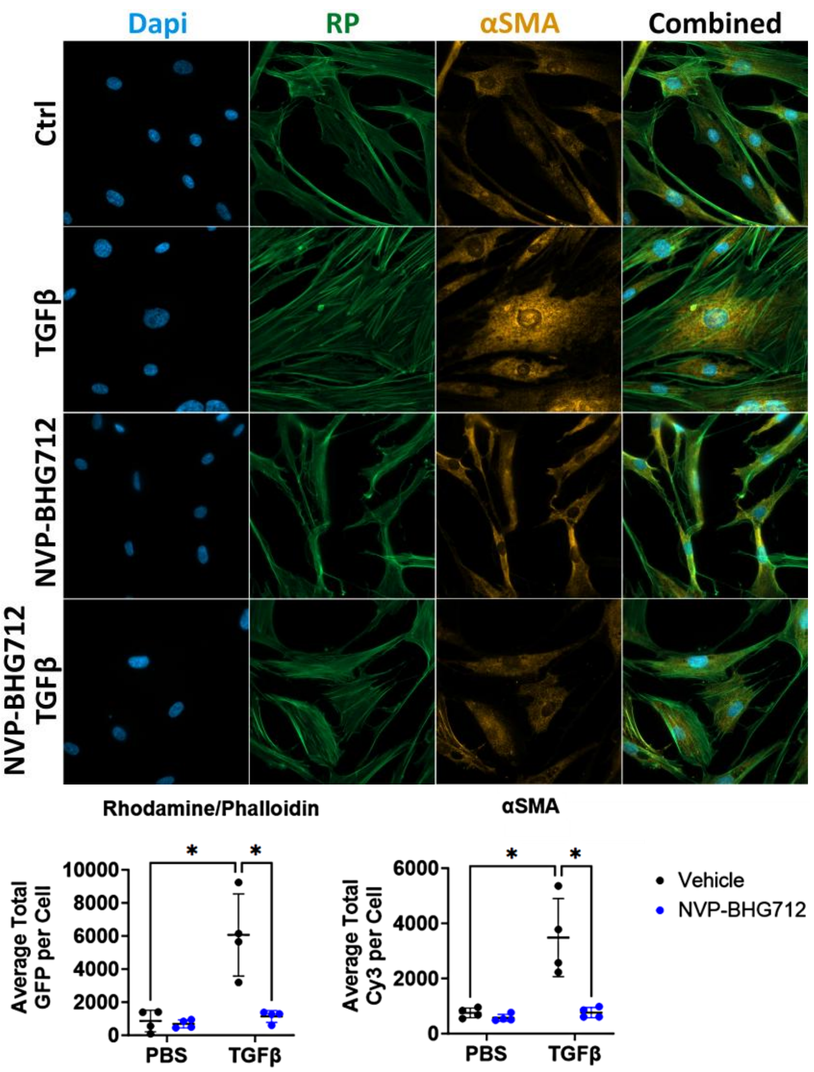

Supplementary Figure 10. Immunofluorescence of α-SMA and rhodamine/phalloidin in human lung fibroblasts from IPF patients. Human IPF fibroblasts (n=4) were cultured and co-treated with NVP-BHG712 (250nM) and TGFβ (20ng/mL) or respective vehicle (as a control) for 24h. Subsequently, cells were immunostained with α-SMA and Rhodamine Phalloidin to observe fibrogenic changes. Imaging was done by confocal microscopy with 63x oil-immersion magnification. Quantification in HALO was performed by averaging total intensity per single cell for 5 cells per patient per group (n=4 patients/group). Data is expressed as mean ± SD. Two-way ANOVA was conducted on log-transformed data to infer statistical significance followed by Tukey's post-hoc tests for multiple comparisons. *, $p < 0.05$.

SUPPLEMENTARY TABLES

The following Supplementary Tables can be accessed in attached supplementary files:

Supplementary Table 4. Pathway and Gene Ontology GSEA results comparing *Ephb4* CKO vs control mice treated with bleomycin**.**

Supplementary Table 6. Genes associated with each fibroblast subcluster identified using single nuclei RNA sequencing.

Supplementary Table 10. PPIs between EphB4 and Elastin in the Integrated Interaction Database ver. 2021-05 (14)(https://ophid.utoronto.ca/iid).

Supplementary Table 1. Fibrotic lobe counts in *Ephb4* CKO mice compared to control mice.

PBS-Challenged Mice				Bleomycin-Challenged Mice			
***Ephb4* Control Animal**	**# of Affected Lobes**	***Ephb4* CKO Animals**	**# of Affected Lobes**	***Ephb4* Control Animal**	**# of Affected Lobes**	***Ephb4* CKO Animals**	**# of Affected Lobes**
Ephb4 Control 1	0/5	*Ephb4* CKO 1	0/5	*Ephb4* Control 1	4/5	*Ephb4* CKO 1	1/5
Ephb4 Control 2	0/5	*Ephb4* CKO 2	0/5	*Ephb4* Control 2	2/5	*Ephb4* CKO 2	0/5
Ephb4 Control 3	0/5	*Ephb4* CKO 3	0/5	*Ephb4* Control 3	4/5	*Ephb4* CKO 3	2/5
Ephb4 Control 4	0/5	*Ephb4* CKO 4	0/5	*Ephb4* Control 4	3/5	*Ephb4* CKO 4	0/5
Ephb4 Control 5	0/5	*Ephb4* CKO 5	0/5	*Ephb4* Control 5	3/5	*Ephb4* CKO 5	2/5
Ephb4 Control 6	0/5	*Ephb4* CKO 6	0/5	*Ephb4* Control 6	5/5	*Ephb4* CKO 6	2/5
Ephb4 Control 7	0/5	*Ephb4* CKO 7	0/5	*Ephb4* Control 7	3/5	*Ephb4* CKO 7	5/5
Ephb4 Control 8	0/5	*Ephb4* CKO 8	0/5	*Ephb4* Control 8	2/5	*Ephb4* CKO 8	0/5

Supplementary Table 2A. Top 50 upregulated DEGs comparing Ephb4 CKO to control mouse fibroblasts following bleomycin treatment.

geneSymbol	baseMean	log2FoldChange	lfcSE	stat	pvalue	padj
Rin2	1.09E+04	1.27E+00	2.39E-01	5.32E+00	1.01E-07	2.73E-04
Ralgapa2	3.57E+03	9.67E-01	1.88E-01	5.16E+00	2.52E-07	4.29E-04
Pank2	3.38E+03	3.98E-01	8.95E-02	4.45E+00	8.63E-06	4.08E-03
Rbm47	1.96E+03	1.40E+00	3.15E-01	4.43E+00	9.24E-06	4.11E-03
Cyb5r4	6.63E+03	1.03E+00	2.35E-01	4.36E+00	1.29E-05	4.80E-03
Ly6i	1.28E+03	4.96E+00	1.15E+00	4.33E+00	1.51E-05	5.15E-03
Cd36	1.26E+05	2.79E+00	6.47E-01	4.30E+00	1.67E-05	5.42E-03
Lpl	9.06E+04	2.77E+00	6.55E-01	4.23E+00	2.35E-05	6.59E-03
Grk3	1.37E+03	1.26E+00	2.98E-01	4.22E+00	2.40E-05	6.63E-03
Cmip	2.44E+03	6.97E-01	1.69E-01	4.12E+00	3.73E-05	8.26E-03
Gm6545	5.50E+02	5.00E+00	1.22E+00	4.11E+00	4.04E-05	8.26E-03
Clcc1	3.00E+03	5.37E-01	1.31E-01	4.09E+00	4.32E-05	8.49E-03
Slc6a12	4.54E+03	2.25E+00	5.64E-01	3.98E+00	6.75E-05	1.09E-02
Usp6nl	3.49E+03	5.19E-01	1.32E-01	3.93E+00	8.52E-05	1.30E-02
A930007I19Rik	1.76E+03	2.34E+00	5.97E-01	3.92E+00	8.68E-05	1.31E-02
Coro2a	6.03E+03	1.62E+00	4.18E-01	3.89E+00	9.99E-05	1.42E-02
Alas1	6.25E+04	2.19E+00	5.64E-01	3.89E+00	1.01E-04	1.42E-02
Pik3cb	5.52E+03	1.14E+00	2.95E-01	3.87E+00	1.07E-04	1.45E-02
Gm13571	3.06E+03	3.82E+00	9.86E-01	3.88E+00	1.06E-04	1.45E-02
Mlxip	3.04E+03	6.25E-01	1.62E-01	3.87E+00	1.10E-04	1.48E-02
Cbl	5.31E+03	9.92E-01	2.57E-01	3.87E+00	1.11E-04	1.48E-02
Lpin2	5.61E+03	9.39E-01	2.43E-01	3.86E+00	1.11E-04	1.48E-02
Gm20056	7.94E+02	3.40E+00	8.95E-01	3.80E+00	1.47E-04	1.65E-02
Zfand5	1.04E+04	7.89E-01	2.08E-01	3.79E+00	1.49E-04	1.66E-02
Sft2d2	5.45E+03	8.43E-01	2.22E-01	3.79E+00	1.50E-04	1.66E-02
Cracr2b	1.09E+03	1.29E+00	3.39E-01	3.79E+00	1.50E-04	1.66E-02
Tmem106c	1.45E+03	9.62E-01	2.55E-01	3.77E+00	1.61E-04	1.72E-02
Ly6c2	9.69E+02	3.57E+00	9.48E-01	3.77E+00	1.62E-04	1.72E-02
Galm	2.19E+03	1.38E+00	3.69E-01	3.75E+00	1.75E-04	1.81E-02
Crybg1	3.75E+03	1.43E+00	3.83E-01	3.75E+00	1.78E-04	1.83E-02
Cd200	1.62E+04	2.13E+00	5.69E-01	3.74E+00	1.87E-04	1.89E-02
Prkcd	2.48E+04	1.11E+00	3.00E-01	3.71E+00	2.06E-04	1.99E-02
Ahcyl2	4.06E+03	9.28E-01	2.51E-01	3.69E+00	2.20E-04	2.05E-02
Tns4	3.78E+02	1.56E+00	4.23E-01	3.68E+00	2.31E-04	2.09E-02
Il1rn	3.62E+04	2.69E+00	7.40E-01	3.64E+00	2.75E-04	2.28E-02
Shtn1	9.94E+03	1.40E+00	3.85E-01	3.63E+00	2.83E-04	2.32E-02
Nostrin	1.06E+03	1.84E+00	5.08E-01	3.62E+00	3.00E-04	2.38E-02
Met	2.74E+04	2.71E+00	7.51E-01	3.61E+00	3.01E-04	2.39E-02
Cdca7l	1.84E+03	1.47E+00	4.07E-01	3.60E+00	3.24E-04	2.45E-02
Ptk2b	8.80E+03	1.14E+00	3.17E-01	3.58E+00	3.38E-04	2.48E-02
Bcl2a1d	1.02E+04	1.76E+00	4.91E-01	3.58E+00	3.44E-04	2.50E-02
Vsig8	1.70E+03	3.28E+00	9.17E-01	3.58E+00	3.45E-04	2.50E-02
Ttc5	2.16E+03	4.71E-01	1.32E-01	3.57E+00	3.54E-04	2.52E-02
Nfya	3.09E+03	7.34E-01	2.06E-01	3.57E+00	3.54E-04	2.52E-02
Gpbp1l1	6.60E+03	4.56E-01	1.28E-01	3.55E+00	3.81E-04	2.63E-02
Chd7	2.67E+03	8.45E-01	2.38E-01	3.55E+00	3.86E-04	2.65E-02
Ezh1	3.36E+03	9.96E-01	2.81E-01	3.55E+00	3.89E-04	2.65E-02
Parp4	1.30E+03	7.59E-01	2.14E-01	3.54E+00	3.97E-04	2.69E-02
Parp6	1.47E+03	6.85E-01	1.94E-01	3.53E+00	4.13E-04	2.69E-02
Prdx1	2.31E+05	2.13E+00	6.03E-01	3.53E+00	4.09E-04	2.69E-02
Hsd17b11	1.39E+03	9.29E-01	2.63E-01	3.52E+00	4.24E-04	2.72E-02

Supplementary Table 2B. Top 50 downregulated DEGs comparing *Ephb4* CKO to control mouse fibroblasts following bleomycin treatment.

geneSymbol	baseMean	log2FoldChange	lfcSE	stat	pvalue	padj
Rnu3a	9.76E+02	-2.65E+01	4.39E+00	-6.03E+00	1.61E-09	1.10E-05
Eln	1.30E+03	-7.53E+00	2.13E+00	-3.53E+00	4.12E-04	2.69E-02
Col11a1	2.92E+03	-5.42E+00	1.70E+00	-3.20E+00	1.39E-03	4.99E-02
Tnfsf15	3.07E+03	-5.29E+00	1.03E+00	-5.13E+00	2.82E-07	4.44E-04
Fibin	8.44E+02	-4.82E+00	1.51E+00	-3.20E+00	1.39E-03	4.98E-02
Nrep	2.56E+03	-4.81E+00	1.19E+00	-4.04E+00	5.32E-05	9.79E-03
Pappa	2.03E+03	-4.79E+00	1.14E+00	-4.19E+00	2.77E-05	7.02E-03
Axin2	2.75E+02	-4.77E+00	1.04E+00	-4.58E+00	4.71E-06	2.83E-03
Timp3	3.70E+04	-4.74E+00	9.40E-01	-5.04E+00	4.57E-07	5.38E-04
Cthrc1	1.11E+03	-4.68E+00	1.44E+00	-3.24E+00	1.18E-03	4.61E-02
Gm21982	3.13E+02	-4.63E+00	1.35E+00	-3.43E+00	5.95E-04	3.24E-02
Hs3st1	4.41E+02	-4.62E+00	1.11E+00	-4.15E+00	3.32E-05	7.53E-03
Cdh6	2.79E+02	-4.55E+00	1.18E+00	-3.87E+00	1.07E-04	1.45E-02
Has2	5.70E+03	-4.52E+00	1.27E+00	-3.56E+00	3.72E-04	2.60E-02
Crispld2	1.93E+03	-4.43E+00	1.33E+00	-3.34E+00	8.40E-04	3.86E-02
Gm21451	3.86E+03	-4.42E+00	1.25E+00	-3.54E+00	3.97E-04	2.69E-02
Loxl2	1.13E+04	-4.36E+00	1.24E+00	-3.51E+00	4.46E-04	2.80E-02
Dkk2	8.53E+02	-4.31E+00	1.23E+00	-3.50E+00	4.61E-04	2.86E-02
Aldh1a2	3.16E+03	-4.15E+00	8.24E-01	-5.04E+00	4.74E-07	5.38E-04
Tll1	6.67E+02	-4.04E+00	1.09E+00	-3.69E+00	2.21E-04	2.05E-02
Fap	2.34E+02	-3.99E+00	1.22E+00	-3.27E+00	1.06E-03	4.40E-02
Nox4	6.87E+02	-3.99E+00	1.18E+00	-3.39E+00	7.08E-04	3.52E-02
Aldh1l2	3.16E+03	-3.67E+00	1.07E+00	-3.42E+00	6.16E-04	3.29E-02
Piezo2	1.67E+03	-3.60E+00	1.08E+00	-3.34E+00	8.42E-04	3.86E-02
Prickle1	9.12E+02	-3.57E+00	1.04E+00	-3.42E+00	6.16E-04	3.29E-02
Wnt5a	1.17E+03	-3.35E+00	1.02E+00	-3.27E+00	1.08E-03	4.43E-02
Csrp2	1.60E+03	-3.33E+00	7.49E-01	-4.45E+00	8.66E-06	4.08E-03
Ramp2	2.58E+02	-3.22E+00	9.18E-01	-3.51E+00	4.53E-04	2.82E-02
Lox	2.43E+04	-3.06E+00	8.13E-01	-3.76E+00	1.67E-04	1.75E-02
Tspan18	3.43E+02	-3.06E+00	9.09E-01	-3.36E+00	7.71E-04	3.67E-02
Ccdc80	7.74E+03	-2.86E+00	6.63E-01	-4.31E+00	1.62E-05	5.39E-03
Pxdn	5.07E+03	-2.83E+00	8.67E-01	-3.27E+00	1.09E-03	4.45E-02
Zfp9	9.10E+02	-2.73E+00	8.49E-01	-3.21E+00	1.32E-03	4.85E-02
Creb3l1	1.28E+03	-2.65E+00	7.55E-01	-3.51E+00	4.42E-04	2.79E-02
Spsb1	3.14E+03	-2.62E+00	7.77E-01	-3.37E+00	7.57E-04	3.64E-02
Mex3b	3.06E+02	-2.61E+00	7.96E-01	-3.28E+00	1.03E-03	4.31E-02
Nxn	3.07E+03	-2.57E+00	8.03E-01	-3.20E+00	1.39E-03	4.98E-02
Lgalsl	1.12E+03	-2.55E+00	6.91E-01	-3.69E+00	2.25E-04	2.06E-02
Lbh	3.07E+03	-2.52E+00	6.62E-01	-3.81E+00	1.37E-04	1.61E-02
Lrig3	6.14E+02	-2.42E+00	4.93E-01	-4.91E+00	9.07E-07	8.06E-04
Zfp449	5.43E+02	-2.17E+00	6.18E-01	-3.51E+00	4.53E-04	2.82E-02
P3h1	3.21E+03	-2.10E+00	5.11E-01	-4.12E+00	3.85E-05	8.26E-03
Crim1	3.38E+03	-2.10E+00	5.98E-01	-3.52E+00	4.33E-04	2.75E-02
Pdia5	6.23E+02	-2.00E+00	5.09E-01	-3.94E+00	8.16E-05	1.27E-02
Nt5dc2	2.36E+03	-1.91E+00	5.94E-01	-3.22E+00	1.29E-03	4.82E-02
Slc7a5	1.76E+03	-1.91E+00	4.77E-01	-4.01E+00	6.12E-05	1.02E-02
Zfp970	5.07E+02	-1.89E+00	5.54E-01	-3.42E+00	6.35E-04	3.35E-02
Dennd5b	6.50E+02	-1.88E+00	5.70E-01	-3.30E+00	9.62E-04	4.17E-02
Csrp1	2.53E+04	-1.86E+00	5.00E-01	-3.73E+00	1.93E-04	1.93E-02
Spry1	7.19E+02	-1.82E+00	4.76E-01	-3.83E+00	1.29E-04	1.56E-02

Supplementary Table 2C. Top 50 DEGs identified in comparison between bleomycin vs PBS challenge in *Ephb4* control mice

geneSymbol	baseMean	log2FoldChange	lfcSE	stat	pvalue	padj
Disp1	918.03	1.31	0.21	6.13	8.91E-10	1.10E-05
Marco	37247.68	-5.56	0.92	-6.04	1.57E-09	1.10E-05
Ralgapa2	3567.91	-1.08	0.19	-5.78	7.50E-09	3.83E-05
Cemip	6761.34	6.67	1.25	5.33	9.72E-08	2.73E-04
Rin2	10929.57	-1.27	0.24	-5.31	1.07E-07	2.73E-04
Aacs	2826.58	1.28	0.24	5.25	1.50E-07	3.26E-04
Nars	16204.76	0.58	0.11	5.24	1.59E-07	3.26E-04
Usp6nl	3491.61	-0.67	0.13	-5.09	3.49E-07	5.09E-04
Aars	18765.35	0.70	0.14	5.05	4.31E-07	5.38E-04
Atf5	2300.43	1.59	0.32	4.96	7.08E-07	7.61E-04
Eno3	796.68	2.16	0.44	4.92	8.68E-07	8.06E-04
Mbd1	4984.71	1.10	0.22	4.91	8.95E-07	8.06E-04
Akt1s1	2217.73	1.05	0.21	4.91	9.01E-07	8.06E-04
Galnt10	2426.05	1.12	0.23	4.84	1.30E-06	1.02E-03
Slc25a4	12978.09	1.00	0.21	4.85	1.26E-06	1.02E-03
Lpl	90620.39	-3.10	0.66	-4.73	2.29E-06	1.67E-03
Akap1	1337.69	1.12	0.25	4.50	6.84E-06	3.88E-03
Epb41l3	2256.18	4.08	0.91	4.48	7.35E-06	3.99E-03
Ccdc80	7744.35	2.97	0.66	4.47	7.66E-06	3.99E-03
Ext2	3728.73	0.64	0.14	4.48	7.56E-06	3.99E-03
Ipo4	3426.73	0.68	0.15	4.44	8.93E-06	4.08E-03
Zfp143	1675.10	-0.42	0.09	-4.45	8.42E-06	4.08E-03
A930007I19Rik	1757.99	-2.65	0.60	-4.44	8.98E-06	4.08E-03
Chpf	1652.09	1.13	0.25	4.43	9.50E-06	4.13E-03
Vsig8	1701.89	-4.04	0.92	-4.40	1.08E-05	4.41E-03
Pmp22	4449.79	2.11	0.48	4.36	1.32E-05	4.80E-03
Il1rn	36150.73	-3.23	0.74	-4.36	1.29E-05	4.80E-03
Ppp1r8	2266.49	0.54	0.12	4.35	1.38E-05	4.95E-03
Aldh1a2	3163.68	3.57	0.82	4.33	1.49E-05	5.15E-03
Ly6i	1278.31	-4.94	1.15	-4.31	1.63E-05	5.39E-03
Wls	15662.32	1.37	0.32	4.27	1.99E-05	5.97E-03
Pop1	1141.08	0.93	0.22	4.27	1.96E-05	5.97E-03
R3hcc1	668.25	0.80	0.19	4.27	1.97E-05	5.97E-03
Arhgef2	8126.47	0.65	0.15	4.25	2.17E-05	6.35E-03
Slc39a6	3655.31	1.05	0.25	4.24	2.23E-05	6.41E-03
Ano8	939.32	0.65	0.15	4.24	2.26E-05	6.42E-03
Mvk	969.95	1.24	0.30	4.19	2.77E-05	7.02E-03
Snta1	812.62	1.07	0.25	4.20	2.69E-05	7.02E-03
Utp4	2725.18	0.56	0.13	4.19	2.82E-05	7.02E-03
Cd200	16157.63	-2.39	0.57	-4.19	2.78E-05	7.02E-03
Sipa1l1	2824.14	1.68	0.40	4.17	3.03E-05	7.26E-03
Lgals1	58452.51	1.14	0.27	4.17	3.11E-05	7.26E-03
Chd7	2668.65	-0.99	0.24	-4.17	3.05E-05	7.26E-03
Gm13571	3060.38	-4.11	0.99	-4.16	3.13E-05	7.26E-03
Tll1	666.53	4.50	1.09	4.11	3.92E-05	8.26E-03
Timp3	36965.03	3.86	0.94	4.11	4.01E-05	8.26E-03
Lrig3	614.49	2.03	0.49	4.12	3.82E-05	8.26E-03
Adpgk	2646.32	0.83	0.20	4.12	3.73E-05	8.26E-03
Acadl	5663.69	-1.09	0.27	-4.11	3.97E-05	8.26E-03
Xxylt1	1144.04	0.76	0.19	4.09	4.23E-05	8.45E-03

Supplementary Table 2D. No genes express significant changes when comparing *Ephb4* CKO and control mice after PBS challenge. Table is the expression of genes related to ECM, protein trafficking, and ER cargo concentration in comparison of Ephb4 CKO and control mice after PBS challenge.

geneSymbol	baseMean	log2FoldChange	lfcSE	stat	pvalue	padj	Gene_group
**Col11a1**	2.92E+03	-1.47E+00	1.62E+00	-9.11E-01	3.62E-01	1.00E+00	ECM
**Eln**	1.30E+03	-2.43E+00	2.03E+00	-1.20E+00	2.31E-01	1.00E+00	ECM
**Itga5**	9.27E+03	1.57E-01	3.64E-01	4.31E-01	6.67E-01	1.00E+00	ECM
**Itgb1**	6.63E+04	-1.50E-01	2.77E-01	-5.41E-01	5.88E-01	1.00E+00	ECM
**Lamc1**	6.49E+03	-9.96E-02	4.19E-01	-2.37E-01	8.12E-01	1.00E+00	ECM
**Lox**	2.43E+04	-7.89E-01	7.73E-01	-1.02E+00	3.08E-01	1.00E+00	ECM
**Loxl2**	1.13E+04	-7.75E-01	1.18E+00	-6.55E-01	5.12E-01	1.00E+00	ECM
**P3h1**	3.21E+03	-4.23E-01	4.87E-01	-8.70E-01	3.84E-01	1.00E+00	ECM
**P4hb**	6.91E+04	-2.03E-01	2.54E-01	-8.01E-01	4.23E-01	1.00E+00	ECM
**Pxdn**	5.07E+03	-7.72E-01	8.24E-01	-9.37E-01	3.49E-01	1.00E+00	ECM
**Tll1**	6.67E+02	3.65E-01	1.04E+00	3.50E-01	7.26E-01	1.00E+00	ECM
**Cnih1**	4.79E+03	-4.24E-02	1.44E-01	-2.95E-01	7.68E-01	1.00E+00	ER Cargo
**Lman1**	1.19E+04	-3.99E-01	2.17E-01	-1.84E+00	6.58E-02	1.00E+00	ER Cargo
**Sec23a**	8.04E+03	-7.46E-01	3.98E-01	-1.87E+00	6.11E-02	1.00E+00	ER Cargo
**Sec24d**	6.86E+03	-6.17E-01	3.41E-01	-1.81E+00	7.01E-02	1.00E+00	ER Cargo
**Arcn1**	1.80E+04	-2.88E-01	1.14E-01	-2.52E+00	1.18E-02	1.00E+00	Protein Transport
**Arf4**	1.64E+04	-3.27E-01	1.65E-01	-1.97E+00	4.83E-02	1.00E+00	Protein Transport
**Copb2**	1.92E+04	-2.83E-01	1.52E-01	-1.86E+00	6.33E-02	1.00E+00	Protein Transport
**Copg1**	1.26E+04	-2.24E-01	1.81E-01	-1.24E+00	2.15E-01	1.00E+00	Protein Transport
**Kdelr2**	8.71E+03	-5.41E-01	2.84E-01	-1.91E+00	5.66E-02	1.00E+00	Protein Transport
**Tuba1a**	2.30E+04	-3.31E-01	4.63E-01	-7.15E-01	4.75E-01	1.00E+00	Protein Transport
**Tubb6**	1.55E+04	2.01E-01	3.72E-01	5.39E-01	5.90E-01	1.00E+00	Protein Transport

Supplementary Table 3. Pathway enrichment analysis of DEGs from RNA sequencing of lung fibroblasts from bleomycin-challenged *EphB4* CKO versus control mice using pathDIP ver. 5 (14)(https://ophid.utoronto.ca/pathDIP).

8 pathways included exclusively in "Upregulated DEGs"
Cytokine Signaling in Immune system
Signaling by Interleukins
Metabolism of lipids
Metabolism of vitamins and cofactors
InlB-mediated entry of Listeria monocytogenes into host cell
Metabolism of water-soluble vitamins and cofactors
Phospholipid metabolism
Effects of PIP2 hydrolysis

11 pathways included exclusively in "Downregulated" DEGs
Post-translational protein modification
Crosslinking of collagen fibrils
COPI-mediated anterograde transport
Collagen formation
COPI-dependent Golgi-to-ER retrograde traffic
Class I MHC mediated antigen processing & presentation
Elastic fibre formation
Extracellular matrix organization
Translation
Cargo concentration in the ER
Fibronectin matrix formation

Pathway Category	Number of Occurrences
Protein Trafficking	**7**
ECM Related	**5**
Lipid Synthesis	**3**
Translation	**3**
MHC Presentation	**2**

10 common pathways in "Downregulated DEGs" and "All DEGs"
Cytosolic tRNA aminoacylation
tRNA Aminoacylation
Metabolism of proteins
ER to Golgi Anterograde Transport
Transport to the Golgi and subsequent modification
Asparagine N-linked glycosylation
COPII-mediated vesicle transport
Membrane Trafficking
Antigen Presentation: Folding, assembly and peptide loading of class I MHC
Vesicle-mediated transport

2 common pathways in "Upregulated DEGs" and "All DEGs"
Metabolism
MET interacts with TNS proteins

Supplementary Table 5. Expression data of NanoString mouse targets after housekeeping gene normalization

Mouse ID	1	2	3	4	5	6	7	8	9
**Genes**	**Vehicle and PBS**								
**Arcn1**	4701.71	6987.46	5932.61	6348.86	5614.27	5880.49	6128.28	6453.05	6495.28
**Arf4**	8122.53	11923.59	10599.98	10215.55	10047.65	8763.44	10695.54	9719.66	6123.83
**Axin2**	128.44	60.33	37.21	44.25	72.88	42.64	37.67	65.13	7.63
**Calu**	15113.83	17449.65	14633.3	15463.92	13394.62	13938.35	15681.3	15052.95	5309.7
**Cdc16**	2071.58	2174.22	2155.49	2308.03	2140.44	2323.28	2163.66	2402.36	1895.41
**Cdc23**	1359.84	1825.63	1384.04	1617.19	1512.88	1431.15	1459.18	1702.19	1399.3
**Cnih1**	6792.15	7861.17	6925.91	7179.58	6370.04	5991.09	6887.16	7130.67	6764.96
**Col11a1**	1662.69	1765.3	1983.74	1420.21	1564.17	1720.97	1501.03	1613.26	2284.67
**Copb2**	4587.41	6308.15	5758	5442.49	5422.63	5298.17	5427.98	5561.25	5136.69
**Copg1**	3314.77	4366.32	3920.25	3713.97	3581.8	3744.43	4046.92	4304.96	3025.03
**Cops3**	2433.34	2786.49	2391.65	2596.35	2525.07	2363.92	2388.26	2518.84	2528.91
**Dda1**	1562.52	1801.05	2062.46	2123.9	1610.05	1724.97	1902.79	2205.71	1480.71
**Eln**	24.75	42.46	120.23	69.94	68.83	175.23	104.63	87.68	198.45
**Fbxw8**	189.72	239.1	229	246.93	245.62	255.18	246.92	234.22	89.05
**Foxk2**	214.46	397.75	246.18	308.31	225.38	197.22	265.05	280.57	129.75
**Itga5**	499.63	4341.74	1152.17	1347.42	600.56	658.27	1569.39	1531.85	618.23
**Itgb1**	36137.2	75928	45626	45110.02	35056.79	34448.12	41562.91	41626.7	28932.37
**Kdelr2**	12826.6	20195.92	16805.96	15492.47	13162.49	13421.99	14449.51	15224.54	14239.75
**Klhl42**	187.36	270.38	154.58	194.12	237.53	169.23	203.67	189.13	142.47
**Lamc1**	1971.42	2366.4	2539.07	2572.09	2643.83	2564.47	2877.9	2960.99	2783.33
**Lgals1**	57411.57	54462.85	65244.4	66568.82	55960.49	60013.54	80762.67	75870.96	49107.68
**Lman1**	3324.19	4180.86	3741.34	3632.61	3409.05	3525.9	3607.49	3976.79	3622.91
**Lox**	46568.17	20575.8	58431.57	30455.4	21999.57	34564.05	34207.02	34185.39	40716.99
**Loxl2**	2823.38	11043.18	5461.72	6415.94	4581.84	4010.94	5397.29	6235.11	2241.42
**Mbd1**	719.99	1888.2	887.39	3334.29	2743.7	2898.94	1498.24	1664.62	2569.62
**Mbtps1**	2280.15	2031.21	1895	2215.25	2363.12	2300.63	2031.13	2139.33	1732.58
**P3h1**	592.72	569.81	797.22	665.15	635.65	640.95	869.09	847.97	664.03
**P4hb**	19245.21	19581.42	18961.45	20924.97	18861.78	21345.93	21519.41	23173.12	20106.62
**Pmm1**	780.08	875.95	865.92	926.35	977.1	975.42	813.29	898.07	1083.82
**Pomgnt1**	361.76	612.27	446.56	431.06	363.04	360.45	527.31	429.62	221.34
**Pxdn**	1168.95	2046.85	1087.76	1530.12	1166.04	1215.28	1372.69	1360.25	407.07
**Rab23**	841.36	1812.23	857.33	1120.47	1056.72	983.41	1177.39	1313.91	798.87
**Rad23b**	3060.24	5354	3583.9	3497.01	3270.04	3033.52	3957.64	3741.32	2933.43
**Sec13**	1797.02	2788.73	2619.22	2208.11	2104	1848.23	2901.62	2459.98	1539.23
**Sec23a**	2888.2	4131.69	3596.78	3252.93	2894.86	2816.99	3719.09	3716.27	3114.07
**Sec24d**	1286.78	2397.68	1764.75	2018.27	1882.67	1816.92	2153.89	2300.9	712.37
**Sec31a**	3293.56	4440.06	4013.28	3892.39	3834.17	3797.07	4137.6	4278.65	3063.19
**Skp2**	71.88	189.94	114.5	139.88	107.97	110.6	139.5	135.27	58.52
**Spsb1**	160.26	741.87	500.94	737.94	516.89	510.36	552.42	655.08	396.89
**Stt3a**	7113.85	8819.79	7814.73	7924.66	7170.34	7672.09	7470.28	8141.47	5495.42
**Tceb1**	6527.01	19152.38	8654.88	9490.46	7218.92	6690.01	8830.41	9092.14	6446.94
**Tfg**	2539.4	3528.36	2932.67	3421.36	3337.52	3229.41	3629.81	3641.11	3348.14
**Tll1**	809.54	692.71	1235.19	396.8	446.71	483.71	398.97	353.21	483.39
**Tuba1a**	23750.13	70947.17	41400.89	42716.34	27856.76	29510.4	42196.25	44114.23	35992.46
**Tubb6**	1229.04	5615.44	2152.63	2643.46	1634.35	1394.5	2690.97	2646.6	2060.78
**UBAC1**	135.51	107.26	123.09	142.74	129.56	119.93	152.06	176.61	117.03
**Uba1**	4819.55	6100.34	5151.14	5365.41	4803.17	5537.36	5418.22	5874.38	5045.1
**Genes**	**Vehicle and TGFβ1**								
**Arcn1**	5174.43	6400.55	6135.6	6505.47	6533.52	7294.82	6299.91	6742.94	6283.01
**Arf4**	8548.71	13064.05	11860.99	11392.2	12116.59	10709.18	11162.21	10807.17	11088.52
**Axin2**	84.33	28.64	29.3	36.34	49.48	48.56	33.33	34.98	33.65
**Calu**	15775.11	16639.87	14046.83	17274.09	16354.52	15280.33	15200.86	14921.92	14677.99
**Cdc16**	2028.55	2525.33	2327.66	2428.71	2419.87	2495.01	2266.64	2549.49	2200.4
**Cdc23**	1051.75	1499.58	1374.8	1465.2	1449.85	1450.19	1424.71	1574	1291.98
**Cnih1**	6456.91	8139.65	7178.71	7350.6	7295.27	7725.15	7246.14	7674.71	7365.43
**Col11a1**	3087.33	4093.91	4936.61	4231.49	4801.76	4175.89	3684.9	3953.46	4302.75
**Copb2**	5044.43	6431.79	6287.97	6164.38	6496.7	6277.66	5734.33	5902.51	5953.29
**Copg1**	3547.61	4238.4	4160.72	4293.61	4409.38	4417.36	4196.72	4327.53	4305.63
**Cops3**	2269.82	2215.53	2368.68	2400.58	2476.25	2633.28	2237.6	2189.03	2336.9
**Dda1**	1784.93	2373.03	2154.2	2167.32	2017.13	2268.37	2067.71	2437.76	2014.87
**Eln**	245.96	1166.34	1252.91	2700.65	3310.49	8505.56	2196.74	1737.23	4561.34
**Fbxw8**	181.54	187.45	225.03	212.16	232.44	194.26	203.22	202.09	217.25
**Foxk2**	238.93	433.47	371.53	365.71	347.5	266.43	368.81	384.76	351.83
**Itga5**	1322.3	5024.63	2212.8	3318.38	2632.74	2970.54	3644.04	4221.63	2353.25
**Itgb1**	47478.76	71670.03	49837.25	60847.87	59989.21	52775.61	52343.36	53691.91	48034.86
**Kdelr2**	17659.6	18731.73	21389.63	20082.58	19988.35	20798.49	16491.17	18622.77	17853.14
**Klhl42**	159.29	179.64	157.05	175.82	156.49	136.25	159.14	141.85	158.61
**Lamc1**	1766.19	2058.02	2048.72	2207.17	2355.43	2119.31	2398.89	2273.56	2361.9
**Lgals1**	62364.91	88164.1	72950.95	78521.66	74888.13	70732.4	88835.33	84655.05	77591.74
**Lman1**	4711.8	4076.98	4894.42	4454.2	4810.97	5116.83	4293.49	4630.67	4918.94

**Lox**	79975.31	41653.7	86364.89	58216.38	56861.68	62192.44	52043.36	52615.37	68993.93
**Loxl2**	4941.36	14078.09	9686.87	12213.88	8803.8	8656.65	7475.17	8970.84	7485.59
**Mbd1**	607.86	1205.39	925.91	1580.07	1415.33	1258.63	993.53	1069.74	1351.58
**Mbtps1**	1923.14	1623.24	1693.59	1821.53	1769.74	1744.95	1624.71	1705.17	1630.35
**P3h1**	941.66	989.31	1026.7	1020.95	1062.07	1197.93	1062.35	1055.16	1082.42
**P4hb**	24563.91	25464.22	23166.44	28592.44	28687.44	35025.23	27918.97	29939.06	27842.9
**Pmm1**	565.7	838.31	866.13	838.09	828.48	832.34	746.23	808.38	835.36
**Pomgnt1**	462.63	607.9	521.56	526.3	451.06	481.6	580.64	561.59	618.11
**Pxdn**	1144.28	2172.57	1454.49	1764.1	1722.56	1739.56	1782.77	1899.49	1482.31
**Rab23**	972.11	1634.96	787.61	1447.61	1380.81	1026.6	1264.5	1275.72	1169.89
**Rad23b**	3685.82	4815.06	3918.11	4288.92	4283.96	4281.11	4554.78	4811.39	4228.73
**Sec13**	2119.9	3310.27	2985.17	2878.82	2948.03	2331.78	3176.3	2882.75	3028.07
**Sec23a**	3356.71	4342.53	4355.28	4068.56	4249.44	4561.71	4061.23	4198.31	3982.64
**Sec24d**	1366.81	1933.05	1763.91	1880.14	1954.99	2041.06	1902.12	2019	1962
**Sec31a**	3560.5	3837.47	4297.85	3973.61	4331.14	4481.44	4111.77	4240.09	4325.82
**Skp2**	85.5	216.09	127.75	151.21	156.49	166.6	192.47	210.84	159.57
**Spsb1**	337.31	1615.43	1051.32	1343.29	1142.62	1147.34	1160.2	1175.64	1052.62
**Stt3a**	8741.96	8759.26	9378.62	9553.08	9917.66	11061.27	8621.39	9517.85	9311.09
**Tceb1**	7364.61	14195.24	9964.64	12808.17	12248.92	10708.51	11063.29	12567.72	9898.44
**Tfg**	2787.49	3630.49	3182.07	3442.63	3461.23	3631.56	3316.08	3553.16	3212.64
**Tll1**	1096.26	1871.87	2400.33	1182.71	930.89	1071.79	1064.5	1079.45	1148.75
**Tuba1a**	29116.43	60174.55	48197.58	48595.31	44336.6	44322.67	47805.79	53547.14	43507.17
**Tubb6**	1657.27	4889.26	3205.51	3436.77	3313.94	2917.93	3399.95	3790.23	2806.98
**UBAC1**	113.61	118.46	159.4	137.14	159.94	108.6	187.09	180.72	150.92
**Uba1**	4526.75	5303.2	4832.3	5294.64	5306.91	6118.47	5388.1	5683.89	5297.69
**Genes**	**NVP-BHG712 and PBS**								
**Arcn1**	3638.85	6292.71	4869.9	5306.5	4655.52	4740.6	5362.05	5881.7	4812.23
**Arf4**	6760.65	11213.36	8140.14	9173.06	8121.29	7190.5	8208.34	8347.55	7116.06
**Axin2**	127.31	55.1	40.82	64.93	77.92	51.68	61.9	51.15	36.53
**Calu**	10864.59	16043.09	11847.62	13248.42	11099.76	11114.19	12624.36	13071.41	11062.53
**Cdc16**	1674.57	2538.85	1882.21	1941.56	1987.58	1909.74	1945.8	2075.19	1893.48
**Cdc23**	1039.3	1591.08	1244.06	1355.91	1392.12	1227.04	1238.03	1354.8	1228.63
**Cnih1**	6451.46	7966.44	6462.05	6601.29	6180.23	6076	5951.87	6585.76	5930.05
**Col11a1**	667.75	1016.64	995.9	887.39	840.86	780.24	768.51	1004.41	1073.98
**Copb2**	3959.74	6092.96	4765.69	5232.65	4597.37	4468.85	4722.02	5196.14	4347.08
**Copg1**	2764.54	3946.72	3191.81	3232.53	2916.83	3166.79	3334.49	3511.61	3495.93
**Cops3**	1886.33	2373.54	2015.43	2243.29	2065.5	1996.44	2126.84	2299.36	1973.84
**Dda1**	1317.31	2489.26	1711.39	1858.8	1758.47	1534.63	1830.18	2191.63	1698.65
**Eln**	23.38	41.33	17.19	39.47	34.89	58.35	35.04	19.59	37.75
**Fbxw8**	189.67	245.21	204.12	235.53	202.36	214.23	218.41	216.55	210.66
**Foxk2**	250.73	427.04	305.11	304.28	274.47	253.41	297.83	294.9	312.94
**Itga5**	281.91	2195.83	820.78	971.42	383.79	496.82	929.69	985.91	444.45
**Itgb1**	28109.2	58076.66	37625.98	37542.71	27315.58	26976.48	31311.54	36471.98	29891.35
**Kdelr2**	8960.07	14085.58	12302.06	11136.26	9609.94	9206.11	9837.63	11702.46	9539.22
**Klhl42**	137.71	267.25	157.93	193.52	203.53	182.56	176.36	193.7	165.6
**Lamc1**	1939.6	2565.02	2127.16	2672.35	2514.42	2419.9	2667.6	2939.22	2986.94
**Lgals1**	65733.19	84415.66	66477.97	67832.27	69722.37	64181.88	72756.21	74459.82	59014.36
**Lman1**	2418.97	3295.13	3031.74	3070.84	2713.3	2862.53	2986.45	3310.29	3224.39
**Lox**	24740.56	16054.11	33722.97	16735.58	11125.34	11199.21	17581.14	21938.04	17717.09
**Loxl2**	1768.11	8184.1	4089.94	5781.38	3625.1	3549.4	4497.77	5093.85	4733.08
**Mbd1**	731.41	2512.67	1241.92	3053.02	2786.57	3816.15	1823.17	1918.49	2201.55
**Mbtps1**	2144.86	2007.11	1797.34	2210.19	2099.23	2138.98	1916.6	2006.63	2031.07
**P3h1**	520.95	786.59	597.32	586.92	552.43	550.17	762.67	686.65	627.1
**P4hb**	15997.44	18404.24	17467.4	15806.18	15283.1	17759.53	17367.4	18592.92	18327.14
**Pmm1**	843.13	1023.53	864.83	1031.25	1123.47	1122.84	836.25	941.29	1274.9
**Pomgnt1**	270.22	577.2	330.89	393.4	327.97	329.27	398.27	350.4	301.98
**Pxdn**	613.19	1666.85	729.46	1231.14	871.09	823.58	907.5	1088.2	864.55
**Rab23**	636.57	1168.17	640.3	975.23	744.33	781.9	847.93	963.05	841.41
**Rad23b**	2830.8	4712.64	3410.97	3454.06	2867.98	2984.23	3753.79	3835.89	3331.54
**Sec13**	1648.59	2967.27	2217.4	2448.27	2081.79	1813.05	2530.95	2305.89	1852.08
**Sec23a**	1886.33	3923.3	2664.32	2779.29	2331.83	2162.32	2662.92	3173.18	2213.72
**Sec24d**	1057.49	2131.09	1477.19	1809.15	1447.95	1486.28	1695.86	1888.02	1726.66
**Sec31a**	2535.89	3988.04	3347.59	3535.54	2870.31	3200.13	3725.76	3938.18	3393.65
**Skp2**	66.26	126.74	98.84	114.58	76.76	100.03	93.44	107.73	68.19
**Spsb1**	133.81	690.16	554.35	453.24	380.3	470.14	416.96	473.37	331.21
**Stt3a**	5556.36	7248.73	6639.31	6257.54	5383.57	6068.5	5996.25	6819.73	6201.59
**Tceb1**	5183.51	11436.52	7519.18	7678.38	5585.93	5949.3	6612.93	7568.4	6057.91
**Tfg**	2170.84	3833.76	2959.76	3250.36	3269.22	3164.29	3399.9	3517.05	3091.66
**Tll1**	455.99	479.39	1046.39	304.28	307.03	511.82	259.28	264.43	312.94
**Tuba1a**	17406.99	59693.92	34986.37	40831.26	31412.87	32365.61	41323.2	44968.61	27412.18
**Tubb6**	700.23	3078.85	1443.89	1554.52	1018.8	956.96	1400.37	1599.65	672.15
**UBAC1**	105.23	185.97	129.99	138.77	184.92	161.72	164.68	162.14	135.16
**Uba1**	4337.78	5513.01	4695.86	4826.52	4136.82	4909.82	5007	5024.2	4882.85
**Genes**	**NVP-BHG712 and TGFβ1**								

**Arcn1**	4659.98	6568.48	5544.66	6096.87	5772.66	6051.22	5735.22	5987.01	5832.21
**Arf4**	9077.15	11523.99	10007.26	10987.73	10114.17	10080.82	9384.67	9065.79	9567.84
**Axin2**	125.83	39.31	50.84	38.53	55.95	37.82	40.29	41.91	34.31
**Calu**	14306.66	15985.52	12254.9	16167.57	14088.58	13400.3	13164.43	12967.14	14136.16
**Cdc16**	2060.68	2490.86	2156.86	2135.9	2052.48	2153.99	2106.34	2225.03	2022.23
**Cdc23**	1022.06	1505.52	1258.78	1442.34	1316.3	1264.76	1266.21	1346.91	1228.44
**Cnih1**	6923.75	7448.99	6793.75	7094.82	6578.53	6710.39	6115	6566.9	6503.62
**Col11a1**	1690.93	3355.65	3243.77	3176.23	3687.79	3209.18	2966.2	2981.32	3742.5
**Copb2**	5373	6358.83	5756.48	6154.67	5668.61	5829.05	5451.46	5497.76	5630.9
**Copg1**	3358.68	4187.68	3779.96	4120.24	3898.83	3913.52	3651.17	3937.41	4265.21
**Cops3**	2059.58	2249.77	2208.91	2290.02	2160.45	2275.31	2064.34	2207.48	2210.95
**Dda1**	1774.81	2365.07	1945.05	2058.83	1877.76	1934.44	1883.45	2128.54	1914.71
**Eln**	132.45	841.21	185.19	1514.26	1658.87	2472.8	1114.47	791.38	2307.03
**Fbxw8**	210.81	230.61	222.71	232.47	202.2	198.01	188.6	197.85	243.63
**Foxk2**	323.4	479.57	347.37	461.09	351.4	388.67	462.08	370.35	407.19
**Itga5**	1209.7	3524.68	1239.41	1988.19	1311.39	1527.38	2005.18	2379.99	1055.72
**Itgb1**	42534.77	62982.94	44481.97	52473.95	46558.18	42857	42834.98	45681.77	43807.33
**Kdelr2**	12954.58	17412.42	17465.51	16222.79	15867.2	15980.77	13515.92	14855.93	14644
**Klhl42**	151.21	220.13	159.77	188.8	192.39	164.4	189.46	158.86	178.43
**Lamc1**	2031.98	2181.63	2035.83	2657.35	2462.78	2334.67	2390.1	2481.35	3275.83
**Lgals1**	76651.33	85377.08	66527.48	78998.62	66567.63	70103.52	72545.83	74074.02	67520.48
**Lman1**	3842.11	4046.17	4243.52	4357.85	4258.08	4385.7	3938.36	4025.13	4972.07
**Lox**	70128.23	36128.58	68637.14	51627.55	45441.14	52058.05	41244.72	44445	64368.18
**Loxl2**	3421.59	11489.92	7452.19	10003.91	6153.51	6561.75	5213.99	6107.86	7403.78
**Mbd1**	639.06	1529.11	1096.59	2229.66	1805.12	1775.82	1183.05	1112.03	1346.25
**Mbtps1**	2142.36	1799.03	1707.82	1891.87	1703.04	1748.5	1593.69	1696.79	1858.67
**P3h1**	1068.42	1025.96	803.68	947.86	969.8	916.53	892.43	936.6	1047.72
**P4hb**	23558.2	24292.75	20144.04	26281.93	24187.05	26939.78	23891.61	24381.74	27718.72
**Pmm1**	683.21	960.44	982.81	932.45	883.42	850.88	750.12	854.73	1040.85
**Pomgnt1**	503.3	545.08	451.46	540.72	385.76	439.1	456.07	423.95	436.93
**Pxdn**	1003.3	2058.46	1143.79	1745.45	1398.75	1289.97	1431.66	1621.75	1071.74
**Rab23**	873.06	1371.87	697.17	1280.51	1053.23	1048.37	1081.03	1079.86	1228.44
**Rad23b**	3827.77	4722.28	3875.58	4285.92	3808.52	4110.48	4382.43	4258.06	4200.01
**Sec13**	2439.26	2967.8	2522.39	3287.97	2699.34	2769.56	2790.46	2462.83	2957.85
**Sec23a**	3334.39	4457.6	3588.72	3880.06	3613.19	3570.02	3554.29	3773.68	3556.06
**Sec24d**	1479.01	2234.04	1594.05	1967.64	1688.31	1744.3	1788.29	1940.44	1860.95
**Sec31a**	3602.6	4170.65	3878	4242.25	3799.69	3843.66	3870.63	4025.13	4126.81
**Skp2**	93.82	170.34	125.88	127.15	126.62	146.02	138.02	131.57	106.37
**Spsb1**	424.94	1396.77	1032.44	1432.07	1056.18	1099.31	969.59	945.37	949.35
**Stt3a**	7407.19	8624.32	8278.87	8598.81	8346.35	9182.14	7702.68	8362.13	8958.2
**Tceb1**	6424.86	11634.05	8753.33	10163.17	8838.12	8784.54	8433.95	9767.51	7320.29
**Tfg**	3112.54	3887.63	3320.02	3645.02	3401.17	3399.84	3356.26	3405.28	3339.88
**Tll1**	803.52	1312.91	1807.07	765.48	616.43	692.26	655.82	736.8	643.96
**Tuba1a**	26649.77	56794.45	45360.69	47259.44	40777.67	43742.55	43935.74	45892.29	26245.51
**Tubb6**	1248.33	3377.92	2362.62	2155.16	2023.03	1996.94	2237.51	2450.16	937.91
**UBAC1**	149	154.61	127.09	208.07	147.24	119.23	174.03	161.78	168.14
**Uba1**	4487.8	5393.15	4773.66	5220.94	4589.86	5514.43	4953.38	5002.66	5452.47

Supplementary Table 7. Characteristics of Human Lung Fibroblast Donors

Cell LOT Number	Sex	Age (years)	Condition
**LOT 70002722**	Male	14	Normal lung
**LOT 70014625**	Female	29	Normal lung
**LOT 70041445**	Female	20	Normal lung
**LOT 19TL148581**	Male	29	Normal lung
**LOT 20TL292907**	Male	37	Normal lung
**LOT 20TL114088**	Male	47	IPF
**LOT 8F5049**	Male	52	IPF
**LOT 8F5060**	Male	55	IPF
**LOT 8F5061**	Male	83	IPF

Supplementary Table 8A. Top 50 DEGs from bulk RNA sequencing comparing primary human lung fibroblasts from control donors to IPF patients.

geneSymbol	baseMean	log2FoldChange	lfcSE	stat	pvalue	padj
AC007952.4	6.34E+04	-6.54E+00	2.16E-01	-3.03E+01	4.64E-202	4.47E-198
NEAT1	1.94E+04	-2.52E+00	1.27E-01	-1.99E+01	9.19E-88	4.43E-84
SNORD3D	3.34E+04	9.38E+00	5.51E-01	1.70E+01	4.64E-65	1.49E-61
MT-ATP6	1.62E+04	-1.84E+00	1.15E-01	-1.60E+01	2.60E-57	6.27E-54
MT-ND5	7.74E+03	-1.63E+00	1.11E-01	-1.47E+01	7.25E-49	1.40E-45
MT-ND4L	3.38E+03	-1.92E+00	1.37E-01	-1.40E+01	1.25E-44	2.01E-41
SNORA53	1.20E+03	-3.43E+00	2.60E-01	-1.32E+01	1.09E-39	1.50E-36
MT-ATP8	2.51E+03	-2.01E+00	1.58E-01	-1.28E+01	2.98E-37	3.59E-34
MT-ND4	1.68E+04	-1.80E+00	1.43E-01	-1.27E+01	1.02E-36	1.10E-33
MT-CYB	1.41E+04	-1.49E+00	1.25E-01	-1.20E+01	4.16E-33	4.01E-30
MTATP6P1	2.77E+03	-1.66E+00	1.52E-01	-1.09E+01	9.11E-28	7.99E-25
MT-ND1	7.99E+03	-1.41E+00	1.35E-01	-1.05E+01	1.44E-25	1.16E-22
TPP1	2.19E+03	-1.29E+00	1.24E-01	-1.04E+01	2.36E-25	1.75E-22
MT-ND2	2.93E+03	-1.78E+00	1.74E-01	-1.02E+01	1.25E-24	8.62E-22
SELENOP	5.68E+03	-3.00E+00	3.10E-01	-9.68E+00	3.78E-22	2.43E-19
AFF4	1.13E+04	-6.36E-01	6.80E-02	-9.34E+00	9.22E-21	5.56E-18
MT-CO2	6.08E+04	-1.21E+00	1.37E-01	-8.81E+00	1.20E-18	6.79E-16
SDCBP	9.48E+03	-9.95E-01	1.13E-01	-8.78E+00	1.57E-18	8.41E-16
LINC00632	4.98E+02	-2.72E+00	3.12E-01	-8.72E+00	2.88E-18	1.46E-15
PDXP	8.61E+02	2.62E+00	3.05E-01	8.58E+00	9.87E-18	4.76E-15
RCN3	1.01E+04	1.31E+00	1.55E-01	8.45E+00	2.95E-17	1.30E-14
RNU2-2P	2.02E+04	-1.72E+00	2.04E-01	-8.45E+00	2.89E-17	1.30E-14
FZD7	4.08E+03	1.30E+00	1.56E-01	8.35E+00	6.65E-17	2.79E-14
WWP1	1.15E+03	-9.58E-01	1.15E-01	-8.34E+00	7.73E-17	3.11E-14
MT-ND3	5.21E+03	-1.30E+00	1.56E-01	-8.29E+00	1.11E-16	4.27E-14
PPDPF	2.31E+03	8.73E-01	1.07E-01	8.18E+00	2.84E-16	1.05E-13
BDNF	3.77E+03	1.13E+00	1.39E-01	8.17E+00	3.07E-16	1.09E-13
SNORD17	5.84E+03	-1.25E+00	1.55E-01	-8.09E+00	5.90E-16	2.03E-13
MT-RNR1	7.89E+05	-1.11E+00	1.39E-01	-8.04E+00	8.64E-16	2.87E-13
SCARNA5	1.77E+03	-1.22E+00	1.52E-01	-8.03E+00	9.37E-16	3.01E-13
H3-2	2.67E+03	1.49E+00	1.89E-01	7.89E+00	3.10E-15	9.64E-13
OTULINL	4.65E+02	2.74E+00	3.51E-01	7.81E+00	5.77E-15	1.74E-12
CENPX	2.25E+03	9.54E-01	1.23E-01	7.78E+00	7.06E-15	2.00E-12
LRBA	4.27E+03	-9.76E-01	1.25E-01	-7.79E+00	6.97E-15	2.00E-12
PAPPA	3.02E+03	-1.86E+00	2.39E-01	-7.76E+00	8.23E-15	2.27E-12
BCAP29	2.15E+03	-8.95E-01	1.16E-01	-7.71E+00	1.28E-14	3.43E-12
C5orf51	2.52E+03	-9.68E-01	1.26E-01	-7.69E+00	1.46E-14	3.80E-12
TPI1	4.76E+04	4.79E-01	6.29E-02	7.62E+00	2.57E-14	6.52E-12
GSK3B	4.39E+03	-5.07E-01	6.66E-02	-7.62E+00	2.64E-14	6.52E-12
MT-ND6	8.06E+02	-1.83E+00	2.42E-01	-7.55E+00	4.40E-14	1.06E-11
EHBP1L1	3.83E+03	9.09E-01	1.23E-01	7.41E+00	1.24E-13	2.92E-11
CA12	3.50E+03	-1.80E+00	2.44E-01	-7.38E+00	1.60E-13	3.67E-11
HEXB	1.28E+04	-7.45E-01	1.01E-01	-7.37E+00	1.73E-13	3.89E-11
TULP4	2.85E+03	-6.78E-01	9.21E-02	-7.36E+00	1.77E-13	3.89E-11
RYBP	1.61E+03	-9.15E-01	1.25E-01	-7.34E+00	2.18E-13	4.67E-11
SEC63	2.13E+03	-1.16E+00	1.58E-01	-7.32E+00	2.47E-13	5.17E-11
MICAL1	4.04E+03	7.12E-01	9.80E-02	7.26E+00	3.75E-13	7.70E-11
RHOQ	6.85E+03	-1.16E+00	1.61E-01	-7.23E+00	4.71E-13	9.30E-11
SEC24B	3.51E+03	-5.43E-01	7.51E-02	-7.23E+00	4.73E-13	9.30E-11
CPD	1.80E+04	-1.09E+00	1.51E-01	-7.21E+00	5.67E-13	1.09E-10
GFPT2	2.16E+03	-9.11E-01	1.27E-01	-7.19E+00	6.49E-13	1.23E-10

Supplementary Table 8B. Top 50 DEGs from RNA sequencing comparing primary human lung fibroblasts from IPF patients treated with NVP-BHG712 compared to vehicle.

geneSymbol	baseMean	log2FoldChange	lfcSE	stat	pvalue	padj
ABHD15	1449.14	3.79E-01	1.05E-01	3.62E+00	2.93E-04	3.66E-02
AC010735.2	743.79	-7.22E-01	1.79E-01	-4.05E+00	5.21E-05	1.11E-02
ADAMTS1	34534.25	-5.63E-01	1.35E-01	-4.17E+00	3.01E-05	7.42E-03
ADM	10737.52	-9.95E-01	2.23E-01	-4.45E+00	8.49E-06	2.63E-03
AHRR	2758.23	-1.13E+00	3.12E-01	-3.63E+00	2.87E-04	3.66E-02
AL109918.1	1124.52	-1.01E+00	1.67E-01	-6.03E+00	1.66E-09	2.66E-06
ANO6	10987.96	3.42E-01	7.33E-02	4.66E+00	3.10E-06	1.24E-03
AOX1	5601.04	-3.08E-01	8.41E-02	-3.66E+00	2.52E-04	3.50E-02
AP000944.5	1236.08	-1.45E+00	4.11E-01	-3.54E+00	4.07E-04	4.71E-02
ARFGAP3	6331.13	-1.81E-01	4.56E-02	-3.98E+00	7.01E-05	1.37E-02
BRD2	13272.91	2.50E-01	5.65E-02	4.42E+00	9.89E-06	2.97E-03
CAMK2G	3208.97	3.22E-01	6.54E-02	4.93E+00	8.42E-07	3.85E-04
CAV1	53700.96	-4.79E-01	1.09E-01	-4.38E+00	1.17E-05	3.30E-03
CHD3	8583.68	-4.14E-01	8.98E-02	-4.61E+00	4.07E-06	1.50E-03
CHKA	823.15	5.63E-01	1.35E-01	4.16E+00	3.13E-05	7.51E-03
CHML	2458.77	-5.38E-01	1.19E-01	-4.52E+00	6.17E-06	2.12E-03
CITED2	16810.67	-6.42E-01	9.52E-02	-6.75E+00	1.52E-11	4.88E-08
CNN2	22849.00	-4.74E-01	1.11E-01	-4.27E+00	1.92E-05	5.13E-03
COL16A1	9510.50	-5.04E-01	1.15E-01	-4.40E+00	1.08E-05	3.14E-03
CORIN	872.68	-1.48E+00	2.84E-01	-5.22E+00	1.80E-07	1.33E-04
CRABP2	1269.05	-1.50E+00	3.00E-01	-5.01E+00	5.53E-07	3.05E-04
CYP2U1	3069.62	3.05E-01	8.62E-02	3.54E+00	3.99E-04	4.68E-02
DPYSL3	16214.37	-3.49E-01	5.92E-02	-5.90E+00	3.71E-09	5.10E-06
DSEL	22244.54	-3.74E-01	7.95E-02	-4.71E+00	2.52E-06	1.10E-03
ELN	3800.96	-7.75E-01	1.84E-01	-4.21E+00	2.60E-05	6.58E-03
ENDOD1	6649.01	4.15E-01	6.49E-02	6.40E+00	1.57E-10	3.78E-07
ETS2	7968.66	-3.33E-01	8.51E-02	-3.91E+00	9.13E-05	1.72E-02
F2R	31246.90	-3.25E-01	8.93E-02	-3.64E+00	2.72E-04	3.58E-02
F2RL2	4062.20	-7.79E-01	2.13E-01	-3.65E+00	2.62E-04	3.58E-02
FAM219A	1512.03	4.32E-01	1.15E-01	3.75E+00	1.76E-04	2.91E-02
FAS	4058.36	-2.78E-01	7.64E-02	-3.63E+00	2.80E-04	3.63E-02
FENDRR	2535.20	-9.25E-01	1.87E-01	-4.94E+00	7.89E-07	3.79E-04
FEZ1	3150.73	2.40E-01	6.08E-02	3.96E+00	7.57E-05	1.45E-02
FOXL1	4729.39	-2.43E-01	6.55E-02	-3.71E+00	2.06E-04	3.20E-02
GALNT5	19937.73	-9.55E-01	1.36E-01	-7.02E+00	2.25E-12	1.08E-08
GARS1	16971.62	-2.54E-01	6.92E-02	-3.67E+00	2.40E-04	3.44E-02
GDE1	7968.85	3.00E-01	6.00E-02	5.00E+00	5.86E-07	3.05E-04
GDF5	1821.18	-1.23E+00	3.37E-01	-3.65E+00	2.65E-04	3.58E-02
GFPT2	1934.82	2.99E-01	7.69E-02	3.89E+00	9.89E-05	1.83E-02
GJA1	6089.47	-7.14E-01	1.42E-01	-5.03E+00	4.87E-07	3.02E-04
GPD1L	1476.13	3.84E-01	9.30E-02	4.13E+00	3.69E-05	8.64E-03
HDGF	25251.47	-2.84E-01	6.26E-02	-4.53E+00	5.77E-06	2.05E-03
HMGCS1	1604.19	-8.45E-01	2.12E-01	-3.99E+00	6.74E-05	1.35E-02
HNRNPL	18040.30	-1.84E-01	5.01E-02	-3.67E+00	2.39E-04	3.44E-02
ICMT	21339.85	2.26E-01	6.29E-02	3.59E+00	3.32E-04	4.08E-02
INSIG1	5636.83	-1.14E+00	2.80E-01	-4.08E+00	4.54E-05	1.04E-02
IRS1	12787.83	-2.73E-01	6.45E-02	-4.24E+00	2.28E-05	5.91E-03
ITPRID2	26802.20	-3.47E-01	4.86E-02	-7.14E+00	9.41E-13	9.04E-09
JARID2	1300.79	-4.19E-01	9.07E-02	-4.62E+00	3.93E-06	1.50E-03
LANCL2	1938.28	-3.75E-01	7.99E-02	-4.69E+00	2.74E-06	1.14E-03
LDLR	13996.78	-5.41E-01	1.47E-01	-3.68E+00	2.36E-04	3.44E-02

Supplementary Table 9. Protein-protein interaction comparison of DEGs identified in lung fibroblasts from bleomycin-treated *Ephb4* CKO mice to human lung fibroblasts from IPF patients treated with EphB4 inhibitor.

uniprot1	uniprot2	symbol1	symbol2	species where gene1 or gene2 is DE	species where protein1 or protein2 is partner	methods	Evidence type
P41250	P41250	GARS1	GARS1	human & mouse	none	Cosedimentation in solution | molecular sieving | x-ray crystallography	exp
P01130	P01130	LDLR	LDLR	human & mouse	none	surface plasmon resonance	exp
P15502	P15502	ELN	ELN	human & mouse	none	atomic force microscopy | biophysical | electron microscopy| light microscopy| transmission electron microscopy	exp
P01130	Q9NVV0	LDLR	TMEM38B	human & mouse	none		ortho

Supplementary Table 11. Gene Ontology enrichment analysis of human DEGs.

GO analysis biological functions associated with differentially-expressed genes upregulated in primary human IPF fibroblasts treated with EphB4 inhibitor compared to DMSO vehicle

Name	P-value	Adjusted p-value	Odds Ratio	Combined score
chloride channel complex (GO:0034707)	0.006235	0.1865	208.03	1056.3
multimeric ribonuclease P complex (GO:0030681)	0.01243	0.1865	92.44	405.55
sarcoplasmic reticulum membrane (GO:0033017)	0.03444	0.1926	30.78	103.7
intercalated disc (GO:0014704)	0.03806	0.1926	27.7	90.54
ion channel complex (GO:0034702)	0.04287	0.1926	24.44	76.97
sarcoplasmic reticulum (GO:0016529)	0.05479	0.1926	18.87	54.82
cell-cell contact zone (GO:0044291)	0.05716	0.1926	18.05	51.66
late endosome (GO:0005770)	0.02311	0.1926	9.2	34.67
autophagosome (GO:0005776)	0.08282	0.2384	12.2	30.39
tertiary granule membrane (GO:0070821)	0.08741	0.2384	11.52	28.07
specific granule membrane (GO:0035579)	0.1078	0.2695	9.21	20.51
mitochondrial outer membrane (GO:0005741)	0.1462	0.3102	6.62	12.72
organelle outer membrane (GO:0031968)	0.1633	0.3102	5.86	10.62
endoplasmic reticulum membrane (GO:0005789)	0.05779	0.1926	3.71	10.56
endocytic vesicle membrane (GO:0030666)	0.18	0.3102	5.26	9.02
specific granule (GO:0042581)	0.182	0.3102	5.19	8.85
tertiary granule (GO:0070820)	0.1861	0.3102	5.06	8.51
endocytic vesicle (GO:0030139)	0.2114	0.3232	4.39	6.81
nuclear membrane (GO:0031965)	0.2262	0.3232	4.06	6.03
axon (GO:0030424)	0.2262	0.3232	4.06	6.03
neuron projection (GO:0043005)	0.1525	0.3102	3.05	5.73
early endosome (GO:0005769)	0.2846	0.3648	3.1	3.89
secretory granule membrane (GO:0030667)	0.2918	0.3648	3.01	3.7
bounding membrane of organelle (GO:0098588)	0.2487	0.3391	2.18	3.04
mitochondrial matrix (GO:0005759)	0.3554	0.4265	2.36	2.44
cytoplasmic vesicle membrane (GO:0030659)	0.3811	0.4398	2.15	2.08
mitochondrial membrane (GO:0031966)	0.4477	0.4974	1.74	1.4
intracellular organelle lumen (GO:0070013)	0.6617	0.709	0.94	0.39
intracellular membrane-bounded organelle (GO:0043231)	0.8164	0.8445	0.71	0.14
nucleus (GO:0005634)	0.8445	0.8445	0.66	0.11

GO analysis biological functions associated with differentially expressed genes downregulated in primary human IPF fibroblasts treated with EphB4 inhibitor NVP-BHg712 (250 nm) compared to DMSO vehicle

Name	P-value	Adjusted p-value	Odds Ratio	Combined score
cortical actin cytoskeleton (GO:0030864)	0.000285	0.02041	26.39	215.47
cortical cytoskeleton (GO:0030863)	0.000739	0.02041	18.7	134.82
caveola (GO:0005901)	0.000816	0.02041	18.04	128.29
plasma membrane raft (GO:0044853)	0.002014	0.03777	13	80.72
Golgi membrane (GO:0000139)	0.003103	0.04655	4.56	26.33
elastic fiber (GO:0071953)	0.01516	0.0788	83.06	347.96
MPP7-DLG1-LIN7 complex (GO:0097025)	0.01516	0.0788	83.06	347.96
CD95 death-inducing signaling complex (GO:0031265)	0.01516	0.0788	83.06	347.96

sarcolemma (GO:0042383)	0.011	0.0788	13.48	60.81
basolateral plasma membrane (GO:0016323)	0.01103	0.0788	6.92	31.17
membrane raft (GO:0045121)	0.01354	0.0788	6.39	27.51
early endosome (GO:0005769)	0.00882	0.0788	5.27	24.93
cell-cell junction (GO:0005911)	0.0094	0.0788	5.17	24.13
actin cytoskeleton (GO:0015629)	0.01576	0.0788	4.41	18.32
bounding membrane of organelle (GO:0098588)	0.008578	0.0788	3.27	15.57
protein kinase complex (GO:1902911)	0.02116	0.08965	55.37	213.49
late endosome (GO:0005770)	0.02002	0.08965	5.49	21.48
nucleus (GO:0005634)	0.02152	0.08965	1.82	6.99
death-inducing signaling complex (GO:0031264)	0.02415	0.09054	47.46	176.71
lipid droplet (GO:0005811)	0.02313	0.09054	8.98	33.82
collagen-containing extracellular matrix (GO:0062023)	0.02866	0.09844	3.65	12.97
intracellular membrane-bounded organelle (GO:0043231)	0.02888	0.09844	1.73	6.13
integral component of plasma membrane (GO:0005887)	0.03132	0.1021	2.22	7.67
platelet dense tubular network (GO:0031094)	0.03305	0.1033	33.22	113.25
early endosome membrane (GO:0031901)	0.0354	0.1062	7.08	23.66
endolysosome membrane (GO:0036020)	0.05062	0.146	20.75	61.91
supramolecular fiber (GO:0099512)	0.05641	0.1567	18.45	53.03
connexin complex (GO:0005922)	0.06216	0.1608	16.6	46.11
adherens junction (GO:0005912)	0.06147	0.1608	5.17	14.41
endoplasmic reticulum membrane (GO:0005789)	0.06565	0.1641	2.43	6.61
gap junction (GO:0005921)	0.07073	0.1658	14.43	38.23
Golgi-associated vesicle membrane (GO:0030660)	0.07073	0.1658	14.43	38.23
endolysosome (GO:0036019)	0.07357	0.1672	13.83	36.09
endosome membrane (GO:0010008)	0.0769	0.1696	3.15	8.08
nuclear inner membrane (GO:0005637)	0.08203	0.172	12.29	30.74
secretory vesicle (GO:0099503)	0.08483	0.172	11.85	29.24
endocytic vesicle membrane (GO:0030666)	0.08391	0.172	4.3	10.65
exocytic vesicle (GO:0070382)	0.08763	0.1729	11.44	27.86
intercalated disc (GO:0014704)	0.09041	0.1739	11.06	26.58
neuromuscular junction (GO:0031594)	0.1097	0.2008	8.96	19.81
cytoplasmic vesicle membrane (GO:0030659)	0.1098	0.2008	2.68	5.93
focal adhesion (GO:0005925)	0.1143	0.2041	2.63	5.71
cell-substrate junction (GO:0030055)	0.1189	0.2073	2.59	5.51
cell-cell contact zone (GO:0044291)	0.1339	0.2183	7.21	14.49
Golgi-associated vesicle (GO:0005798)	0.1339	0.2183	7.21	14.49
axon (GO:0030424)	0.1284	0.2183	3.31	6.8
intracellular non-membrane-bounded organelle (GO:0043232)	0.1404	0.224	1.78	3.49
tertiary granule lumen (GO:1904724)	0.1548	0.2419	6.14	11.45
specific granule lumen (GO:0035580)	0.1728	0.2645	5.43	9.54
late endosome membrane (GO:0031902)	0.1878	0.2799	4.94	8.27
clathrin-coated endocytic vesicle membrane (GO:0030669)	0.1903	0.2799	4.87	8.08
clathrin-coated endocytic vesicle (GO:0045334)	0.2291	0.3225	3.94	5.81
tight junction (GO:0070160)	0.2291	0.3225	3.94	5.81
azurophil granule lumen (GO:0035578)	0.2408	0.3225	3.72	5.29
clathrin-coated vesicle membrane (GO:0030665)	0.2408	0.3225	3.72	5.29
neuron projection (GO:0043005)	0.2404	0.3225	1.81	2.58
apical junction complex (GO:0043296)	0.2592	0.3295	3.41	4.6
secretory granule lumen (GO:0034774)	0.2508	0.3295	2.12	2.93
intracellular organelle lumen (GO:0070013)	0.2589	0.3295	1.59	2.15
cytoskeleton (GO:0005856)	0.2766	0.3458	1.68	2.15
mitochondrial matrix (GO:0005759)	0.287	0.3529	1.92	2.4
azurophil granule (GO:0042582)	0.3783	0.4561	2.14	2.08
specific granule (GO:0042581)	0.3878	0.4561	2.07	1.96
vacuolar lumen (GO:0005775)	0.3897	0.4561	2.06	1.94
tertiary granule (GO:0070820)	0.3953	0.4561	2.02	1.88
endocytic vesicle (GO:0030139)	0.4401	0.5002	1.75	1.44
nuclear membrane (GO:0031965)	0.4655	0.521	1.62	1.24
lytic vacuole (GO:0000323)	0.4896	0.54	1.51	1.08
dendrite (GO:0030425)	0.5641	0.6132	1.22	0.7
endoplasmic reticulum lumen (GO:0005788)	0.5839	0.6256	1.15	0.62
lysosomal membrane (GO:0005765)	0.6381	0.6741	0.99	0.45
organelle inner membrane (GO:0019866)	0.6557	0.6778	0.95	0.4
nucleolus (GO:0005730)	0.66	0.6778	0.89	0.37
nuclear lumen (GO:0031981)	0.6688	0.6778	0.88	0.35
lysosome (GO:0005764)	0.7712	0.7712	0.68	0.18

Supplementary Table 12. Mouse genotyping primer sequences.

Gene Name	Forward Sequence	Reverse Sequence
Cre	GCATTACCGGTCGATGCAACGAGTGATGAG	GAGTGAACGAACCTGGTCGAAATCAGTGCG
Ephb4	GCCCTTAAAGGACCGACTTC	GCCTAACGCTGGAGAAAGTG

Supplementary Table 13. RT-qPCR primer sequences.

**Mouse**		
**Gene**	**Forward Sequence**	**Reverse Sequence**
Ephb4	CTACGTCTCTAACCTCCCATCT	GCTGGTCACCCTTTCTCTTT
Col1a1	CCGTGCTTCTCAGAACATCA	GAGCAGCCATCGACTAGGAC
Acta2	CTGACAGAGGCACCACTGAA	CATCTCCAGAGTCCAGCACA
**Human**		
**Gene**	**Forward Sequence**	**Reverse Sequence**
COL1A1	GAACGCGTGTCATCCCTTGT	GAACGAGGTAGTCTTTCAGCAACA
ACTA2	TTCAATGTCCCAGCCATGTA	GAAGGAATAGCCACGCTCAG

Supplementary Table 14. Primers used for mouse NanoString CodeSet

Gene	Accession#	Sequence
**Acad9**	NM_172678.3	TGCAAAAACCGAGGTGGTCGATTCTGATGGTTCGAAAACAGACAAAATGACCGCATTCATAGTAGAAAGAGACTTCGGCGGAATCACTAATGGGAAACCT
**Arcn1**	NM_145985.4	TGCTTTTCCCTTCATCAGTCTTCTTTCCAGCAGAAATGAGTCTAGTGAGTCTGTGCCATTTCTCATTAGCCCTAAATCACTGGAGAGATCCATCTTTACC
**Arf4**	NM_007479.3	GTTGGTGGTCAAGATAAAATTAGGCCTCTCTGGAGGCATTACTTCCAGAATACCCAGGGTCTCATTTTTGTGGTAGATAGCAATGATCGTGAAAGAATCC
**Axin2**	NM_015732.4	ACCGCGAGTGTGAGATCCACGGAAACAGCTGAAAACGGATTCAGGTCCTTCAAGAGAAGCGACCCAGTCAATCCTTATCACGTAGGTTCCGGCTATGTCT
**Calu**	NM_184053.3	GTGGGCGGCCTGGTGTGGTAAAAGCCCAGTTGTGGTGTGACTTCACCTTAGCCATTGCATCAAGCTCTTGATAGCAGATACACTCTTACGTTTCTAACCC
**Cdc16**	NM_027276.2	CTGGGAGATGTCACAGTCTTCCATAAAGAGCTCGATTTGTCTCCTGAGAGGGAAGATCTATGATGCTTTAGATAATCGCACCCTGGCTACCTACAGTTAC
**Cdc23**	NM_178347.4	TATTATTATAGACGGGCCCACCAGCTTCGTCCCAATGATTCTCGCATGCTGGTTGCCTTAGGAGAATGTTATGAGAAACTCAATCAACTAGTGGAAGCCA
**Cnih1**	NM_009919.2	CTTTTGGCATACCATATTTGGAGGTATATGAGTAGACCAGTGATGAGCGGCCCTGGCCTCTATGACCCGACGACCATCATGAATGCAGACATTCTAGCCT
**Col11a1**	NM_007729.2	GTAAAGGGAGCAGATGGTGTCAGAGGTCTCAAGGGCTCTAAAGGCGAAAAGGGTGAAGATGGCTTTCCAGGATTCAAAGGTGACATGGGTCTTAAAGGTG
**Copb2**	NM_015827.2	TCTGTGAACAGATTAAACTATTACGTGGGAGGAGAGATAGTCACCATCGCCCACCTGGACAGGACAATGTATCTTCTGGGCTATATTCCAAAAGACAATA
**Copg1**	NM_017477.2	GCAACTGAGGCTTTCTTTGCCATGACCAAGCTCTTCCAGTCCAATGATCCCACACTCCGCCGCATGTGCTATTTGACCATCAAGGAGATGTCCTGCATCG
**Cops3**	NM_011991.1	CACTTTCTGTGCTACTACTATTATGGAGGAATGATCTATACGGGGCTGAAGAACTTTGAAAGAGCGCTGTACTTTTATGAGCAGGCTATAACTACTCCTG
**Dda1**	NM_001294258.1	CCCTTTCAGATACAGGCAGAAGTTTCTCCCACTCTCAGGTCTTGGTCTGAATTCCAACTGCCTTCCTATGATTGCCTTTTGCTGTGCTACTACTGTGCAT
**Eln**	NM_007925.3	GTCCCTGCCTCCTGTTACCTAAAGCTACTTCCCACATCTGGGACACCCTGGAGTCAGATGGCTCCTCACACTGGGAATAGCTCCCTTGTTCTTATGGAAT
**Tceb1**	NM_001310470.1	ACTAACAGCTCCACTGAAATTCCTGAATTCCCAATTGCACCTGAAATTGCACTGGAACTGCTGATGGCTGCAAACTTCCTAGATTGTTAAATAAAATAAA
**Fbxw8**	NM_172721.2	ACATCTGCAGCTTGCCTTTTGCCTTAAGCGGCCACTTCTGCTCTGTTATTAAAGGTTCTACACTGATGAGCGTGTGTGCTGCTCCTGTAAGGACTCGTTT
**Foxk2**	NM_001080932.2	TTTGGGTGGCTCTCCCTTAGGACACCTACCTGCCCGTTTCCGTTTAGAGCAGTTACCTGATGAATTTGACCTACCTTGTGCCATTGTAGAGGGTGGGTGG
**Itga5**	NM_001314041.1	CCTCAGCAAGAACCTGAACAACTCACAAAGCAACGTGGTCTCCTTCCCACTCTCGGTGGAGGCTCAAGCCCAGGTCTCCCTTAATGGTGTCTCCAAGCCT

**Itgb1**	NM_010578.1	CTGTGATAGGTCTAATGGCTTAATTTGTGGAGGCAATGGCGTGTGCAGGTGTCGTGTTTGTGAATGCTATCCCAATTACACTGGCAGTGCATGTGACTGT
**Kdelr2**	NM_025841.4	AAAGAGTCCTCGTTGTCCTGGAAACCCTTCCTCAGATGTCACACTACATGTCAGGTTCGGGAGGATGACTAGAAAGTCCTAAGGTTTCATTACCAAAACT
**Klhl42**	NM_001081237.1	AGACCTTGACCCTGCTGTGTTCTGTTGTTTCTAACGCCAAGAACCCAAGCAAACTTAAATGCCTTAGGTTTCCGTTCAAGCAGAGGGAAGGCGAGGATAC
**Lamc1**	NM_010683.2	GTTCTCAAGTCTTACTATTACGCAATCTCAGACTTTGCTGTGGGCGGCAGGTGTAAATGTAACGGACATGCCAGCGAGTGTGTAAAGAACGAGTTTGACA
**Lgals1**	NM_008495.2	TCTCAGGAATCTCTTCGCTTCAGCTTCAATCATGGCCTGTGGTCTGGTCGCCAGCAACCTGAATCTCAAACCTGGGGAATGTCTCAAAGTTCGGGGAGAG
**Lman1**	NM_027400.3	GGACATCAAGGAACACCTGCACGTGGTGAAGAGAGATATCGACAGCCTCGCACAGCGCAGCATGCCATCTAATGAAAAACCAAAATGCCCAGACCTACCA
**Lox**	NM_010728.2	GATACTTGTTGCTATTCGATCCCACGCTGCTTAGCTTTTCTGTGGGCAGAAATGTCTAATGTGACAATCAGCACATCCCCATTGTGAGGTTTCACGGCAT
**Loxl2**	NM_033325.2	TTCCACTTGGCGCTCTGGTCTTCCAAACTCCCACCACAATAACCCATTCAGTTCTGTTCTCTAAAAGCGCTCTAGGGCTTCTGGACCCAAAGTTCTAAGT
**Mbd1**	NM_013594.2	GTGTGCTGTGAGAATTGTGGAATCCACTTTTCATGGGATGGTGTCAAAAGGCAGAGACTTAAGACGTTGTGCAAAGATTGTCGAGCACAGAGAATCGCCT
**Mbtps1**	NM_019709.3	TGCTGTGGCAGATGGGATACACAGGTGCTAATGTCAGAGTTGCTGTTTTTGATACTGGGCTCAGTGAGAAGCATCCGCATTTTAAGAATGTGAAGGAGAG
**P3h1**	NM_019782.3	TGCTGTTGTACGGAGTTATAGGAACCTGCGGCTGAGTAGTCATTGGTCAGTCGAACTATTTATTTAGTGAGCACCAGCTTGTTGCTGGGAATATGAGAGG
**P4hb**	NM_011032.2	AAGTGACGGTCATCGGTTTCTTCAAGGACGTAGAGTCAGACTCTGCCAAGCAGTTCTTGCTGGCAGCAGAGGCTATTGATGACATACCTTTTGGAATCAC
**Pmm1**	NM_013872.4	CCTGGAGGAGAGGATCGAGTTCTCGGAACTGGACAAGAAGGAGAAGATCCGGGAGAAGTTTGTGGAAGCCTTGAAGACAGAGTTTGCTGGCAAGGGGCTG
**Pomgnt1**	NM_026651.2	CTCTCTGCTCTCAGCCCAAGGGGTATCTCCACAGATGATAACGGTCTTCATTGATGGCTACTATGAGGAGCCAATGGATGTGGTGGCGCTGTTTGGTCTG
**Pxdn**	NM_181395.2	GGCTTATGTTGGCACTAAGGCCATTGTGGAGTGTACTTATATGATCCCTATGCTGATAGGATTACCTTCCTAGACATAGCTAGACGCAAAGCCACATGTG
**Rab23**	NM_008999.3	TAGTAACAAGATTGGTGTCTTTAATGCATCGGTCAGAAGTCACTTGGGCCAGAATTCAAGTTCCCTTAATGGTGGCGATGTCATCAACCTTAGACCTAAC
**Rad23b**	NM_009011.4	ACCTCACACTCACACCAGTGCATTACACTAACCTTGTTCACTGGATTGTCTGGGGTGACTTGGGCTCATATCCACAATATTTGGTATACGGTAGTAGGTT
**Rplp0**	NM_007475.5	TCAGAACACTGGTCTAGGACCCGAGAAGACCTCCTTCTTCCAGGCTTTGGGCATCACCACGAAAATCTCCAGAGGCACCATTGAAATTCTGAGTGATGTG
**Sec13**	NM_024206.4	TGACCGGAAAGTCATCATCTGGAAGGAGGAAAACGGCACTTGGGAGAAGACCCATGAGCACTCGGGACACGACTCCTCAGTGAACTCTGTTTGCTGGGCC
**Sec23a**	NM_009147.2	TCAGGAGCTATTGGACCCTGTGTGTCTCTTAATTCAAAAGGACCTTGCGTGTCTGAAAATGAGATTGGAACAGGAGGCACTTGTCAGTGGAAAATCTGTG

**Sec24d**	NM_027135.2	TCTTATGGTTTGGAGTGGGCAGCCCACCAGAGCTGATTCAGGG AATATTTAATGTGCCATCGTTTGCACATATCAACACAGATATGA CATCGCTGCCTGA
**Sec31a**	NM_026969.1	ATTTACCTAGCCACAGGAACATCCGCTCAGCAGTTGGATGCGA CGTTTAGTACCAATGCTTCCCTCGAGATATTCGAGCTGGACCTT TCGGACCCATCCT
**Skp2**	NM_013787.2	CTTCAGCTCTTTCCGGGTACAGCACATGGACCTGTCGAACTCAG TGATAAATGTGTCGAACCTCCATAAGATTCTGTCCGAGTGCTCC AAGCTGCAGAAT
**Spsb1**	NM_029035.2	CCCAGGTTGGACTTCCCTTGGGCCAACGAGTGCCAGCTTTAATG TCAGCTGCCGGTGCTCTGTGGCCTGTATTTATTCTTTAAACAGT AGCAAAGGCCAT
**Stt3a**	NM_008408.4	ATGGTCTCTTCATGGGGAGGCTATGTGTTCCTGATCAACTTGAT TCCTCTACATGTCCTGGTGCTAATGCTGACAGGCCGTTTTTCTC ACCGGATCTACG
**Tfg**	NM_001252443.1	TGATACTGTGGATGGCAGGGAAGAGAAGCCTGCCGCTTCTGAC TCTTCTGGGAAACAGTCAACTCAGGTTATGGCAGCAAGTATGT CAGCTTTTGATCCT
**Tll1**	NM_009390.2	CCTGTACTCCCATGCTCAGTTTGGTGATAATAACTACCCAGGAC AACTGGACTGTGAATGGTTGTTGGTGTCAGAACGAGGATCTCG ACTTGAATTGTCC
**Tuba1a**	NM_011653.2	GGGAGGAAGAAGGAGAGGAATACTAAATTAAATGTCACAAGG TGCTGCTTCCACAGGGATGTTTATTGTGTTCCAACACAGAAAGT TGTGGTCTGATCAG
**Tubb6**	NM_026473.2	CAGGACGCCACGGTCAATGATGGGGAAGAGGCATTTGAAGAC GAGGATGAAGAAGAGATCAACGAATAGGGAGCCATAAGATGC TACAGTGAACGTCTGC
**Uba1**	NM_001136085.1	ACTGGTGACTTCGTCTCATTCTCAGAAGTACAGGGCATGATCCA ACTCAATGGATGTCAGCCCATGGAGATCAAAGTGCTGGGTCCT TATACCTTTAGTA
**UBAC1**	NM_001362681.1	AGAGCCTCAAAGGCCCTTCGGCTGAACCACATGTCAGTGCCTC AGGCCATGGAGTGGCTAATTGAACACTCAGAAGATCCAGCTAT TGACACACCTCTTC

Supplementary Methods

Immunohistochemistry

Lung sections (4 µm) were deparaffinized in xylene followed by rehydration using a graded series of alcohol washes. Antigen retrieval was performed using proteinase K (20 ug/mL) for 15 min, and endogenous peroxide was blocked for 30 min using 3% H_2O_2. Nonspecific IgG binding was blocked by incubating sections with 2% goat serum in PBS for 30 min. Sections were then incubated with primary antibodies for αSMA (Sigma-Aldrich; A2547) at 1:1000 overnight at 4°C. Sections were incubated with biotinylated secondary anti-mouse antibody (Vector Laboratories; BA-9200) for 30 min, followed by incubation using the Vectastain Elite ABC kit (Vector Laboratories), as per manufacturer's direction, and counterstained with Harris hematoxylin. Lung sections were assigned αSMA positivity expression scores on a scale of 0–3 (absent, mild, moderate, or severe staining) based on the level of brown staining of αSMA against the blue counterstain.

Western blot

Protein lysates were generated from mouse lung fibroblasts using RIPA buffer with protease and phosphatase inhibitor tablets. Lysates were applied to SDS-acrylamide gels (7.5 or 10%) for electrophoresis. Electroblotting was performed on PVDF membranes and blocked with 1% TWEEN20 10 mM Tris-buffered saline (TTBS) containing 2.5% skim milk, and 2.5% bovine serum albumin (BSA). Probing was performed for EphB4 (R&D, catalog #AF446-SP) at 1:500, phospho-EphB4 (ThermoFisher, catalog #PA5-64792) at 1:500, elastin (Abcam, ab213720) at 1:300, phospho-SMAD 2/3 (Abcam, catalog #ab272332), SMAD 2/3 (Abcam, catalog #ab217553), and GAPDH (Abcam, catalog #ab9485) at 1:1000 for 2 h. Membranes were washed with TTBS 3 times for 5 min each and incubated in HRP-conjugated anti-rabbit (1:5,000; Sigma-Aldrich, catalog #SAB3700843) or anti-mouse (1:10,000; Sigma-Aldrich, catalog #A2179) secondary antibodies for 1 h at room temperature. Membranes were washed

multiple times in TTBS before visualizing with chemiluminescence substrate (Clarify Western ECL Substrate, BIO-RAD, or SuperSignal West Pico, Thermo Science) and scanned using a BIO-RAD Chemidoc apparatus. Membrane signal intensity was quantified using ImageJ (National Institutes of Health, USA).

Immunofluorescence

Human IPF fibroblasts (n=4) were cultured in 6-well plates with 150,000 cells placed on coverslips (22mm x 22mm) inside each well. When cells reached 80% confluency, they were co-treated with NVP-BHG712 (250 nM) and TGFβ1 (20 ng/mL), or DMSO and HCl as a control, for 24h. Cells were fixed in 4% paraformaldehyde. Fixed cells were permeabilized in 0.2% Triton-X and blocked with 0.5% BSA in PBS. Subsequently, cells were immunostained in the following order in the dark room. Cells were first incubated with primary anti-αSMA (Sigma, A2547; clone 1A4) in a 1:400 dilution, followed by secondary anti-mouse (Sigma, red channel, AF568) in a 1:300 dilution. Lastly, 3.5µL of Rhodamine Phalloidin (Sigma, A11055) counterstain in 500µL PBS and DAPI (1µg/mL) were applied. Imaging was done by confocal microscopy with 63x oil-immersion magnification using Zen Blue v3.12 (Zeiss). HALO v4.1 (Indica Labs) was utilized for the area quantification of fluorescence stains. To ensure the specificity in analyzing the expression of fibrotic markers, artificial lines were drawn to produce layers to capture single cells for the consecutive quantification. The five most representative single cell layers per treatment for each cell line, resulting in a total of 80 quantification data points, were obtained.

Flow cytometry

Lungs were recovered from *Ephb4* control and *Ephb4* CKO mice after vascular perfusion with PBS, then minced and digested for 30 min at 37°C in 0.5 mg/ml collagenase VIII (Sigma). Single cells were filtered through a 70 µm filter and 1 x 10^6 cells were labeled with a live/dead marker (eFlour780, Fisher) in PBS according to the manufacturer's instructions. Cells were

washed once, resuspended in 50 µl of FACS buffer and labeled with CD45-PE-Cy7 (Biolegend, 30F11), F4/80-APC (Biolegend, BM8), CD31-PE (Biolegend, 390) and goat anti-EphB4 (R&D, AF446), followed by donkey anti-goat-AF488 secondary (Invitrogen, A11055). At least 100,000 events were acquired using a BD FACS-Canto II flow cytometer. Single cells, live cells, and either macrophages ($CD45^+$/$F4/80^+$) or endothelial cells ($CD45^-$ /$CD31^+$) were gated. Geometric mean fluorescence intensity (gMFI) of EphB4 was determined for each gated population using FlowJo_v10.8.4. Cell sorting of similarly labeled cell populations was performed on a BD FACS ARIA III cell sorter, and cells were lysed in RIPA buffer for Western blot analysis. Total EphB4 was detected by incubating the membrane with an anti-EphB4 antibody (R&D, AF446) at 1/250 dilution, followed by a donkey anti-goat HRP secondary antibody (1:10,000).

RNA isolation and qRT-PCR

RNA isolation was performed using the TRIzol-chloroform method. Nanodrop was used to evaluate the quality of the RNA, and cDNA was generated using the QuantiTect™ Reverse Transcription kit (catalog 205311; Qiagen) according to the manufacturer's instructions. qRT-PCR was performed using SYBR™ Green PCR Master Mix (catalog 4309155; ThermoFisher) according to the manufacturer's instructions. qRT-PCR primer sequences can be found in **Supplementary Table 13**. CT values generated from qRT-PCR were analyzed according to the ΔCT method and expressed as relative expression ($2^{-\Delta CT}$).
