# Supplementary material for: Identification of Ephrin type-B receptor 4 as a critical mediator of tissue fibrosis
Source: JCI Insight. 2025 Dec 22;10(24):e189156. doi: 10.1172/jci.insight.189156 (PMC12890498; doi:10.1172/jci.insight.189156)

Source Images for “Identification of Ephrin type-B receptor 4 as a critical mediator of tissue fibrosis”

Figure 2A

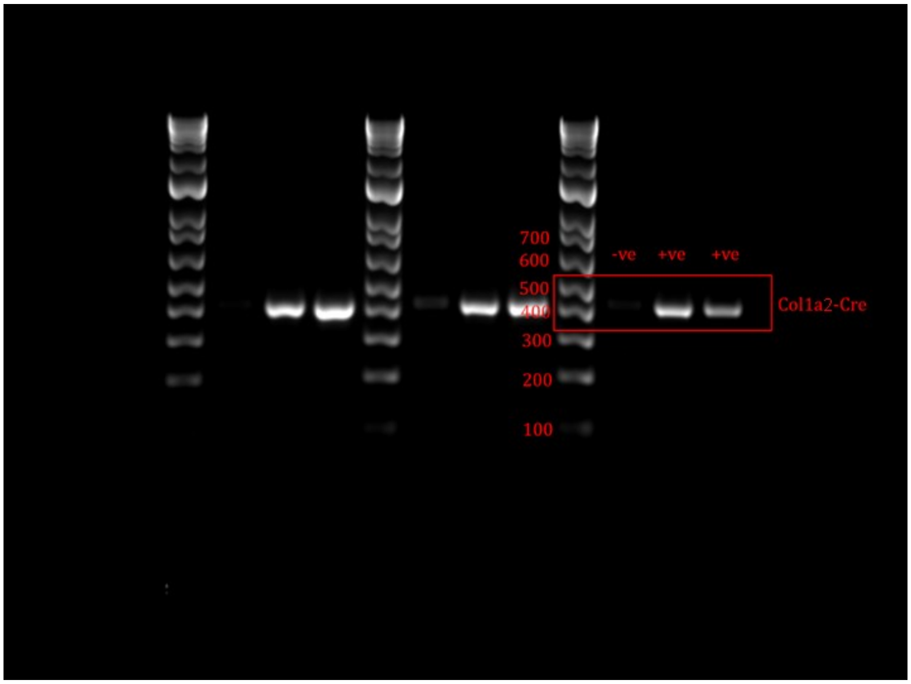

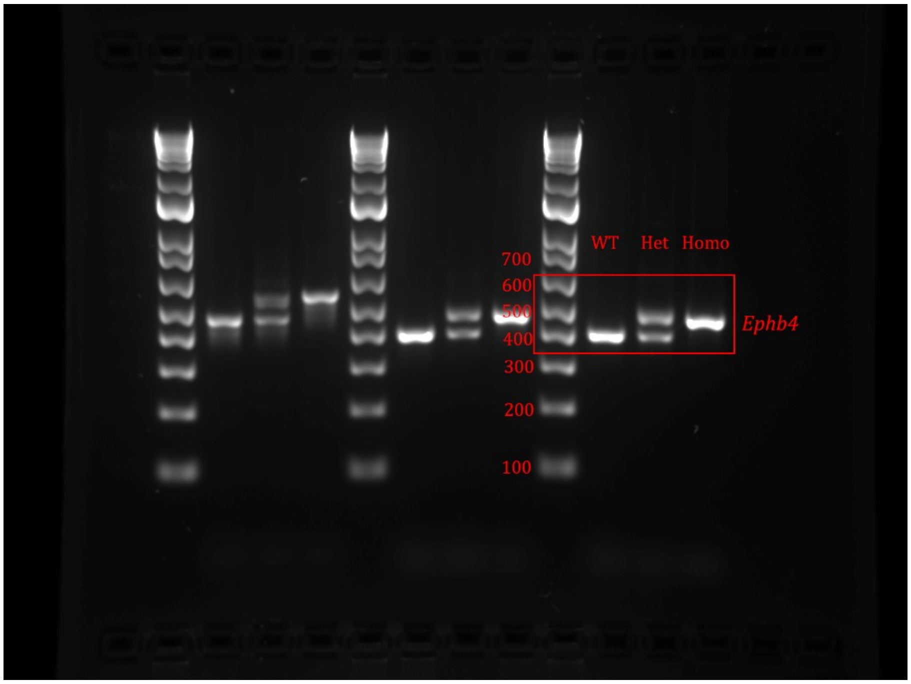

Figure 2B

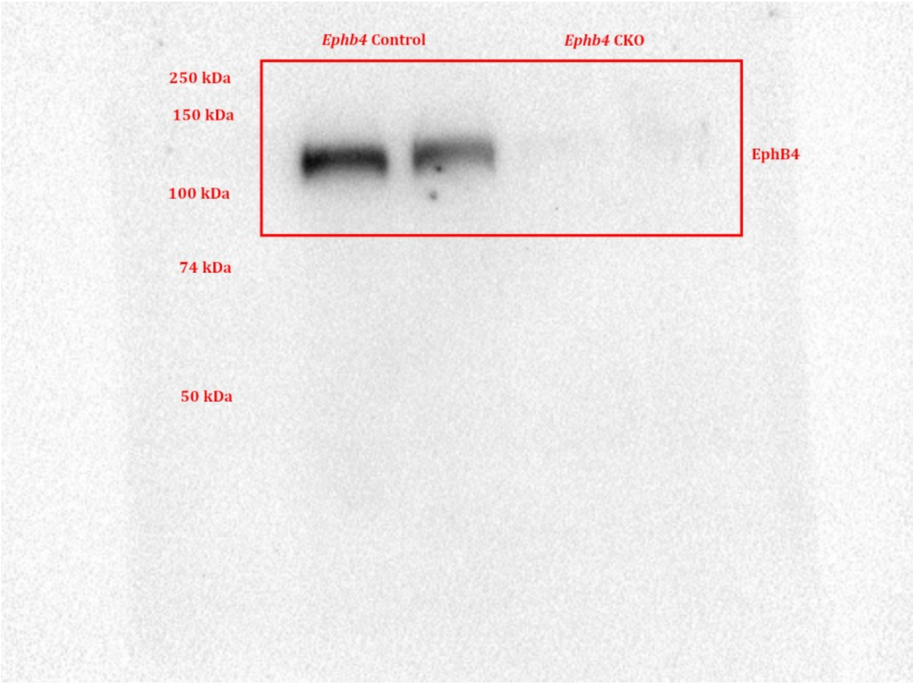

Ephb4 Control
Ephb4 CKO
250 kDa
150 kDa
100 kDa
74 kDa
50 kDa
EphB4

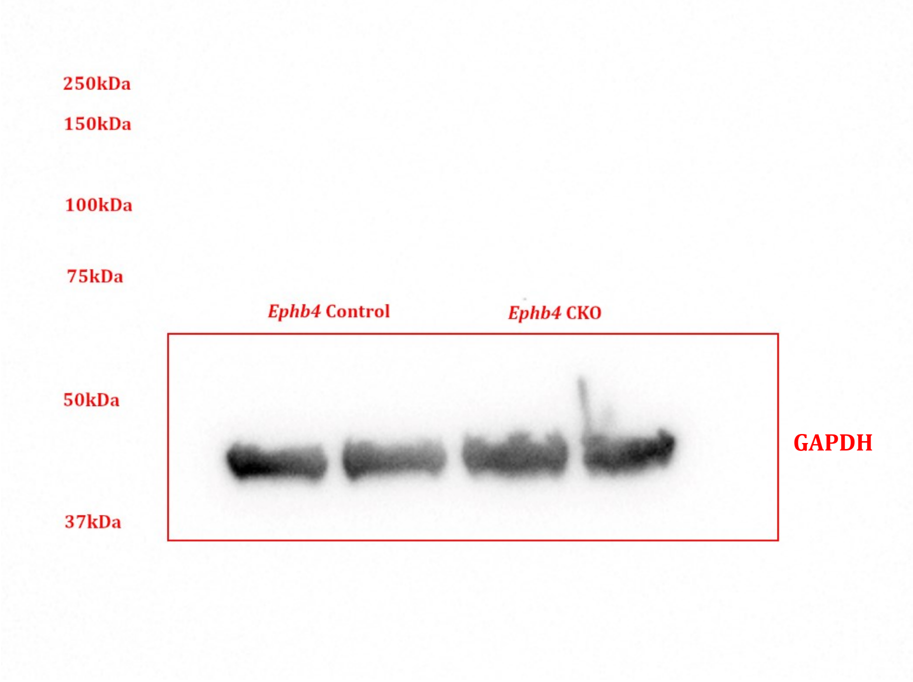

250kDa
150kDa
100kDa
75kDa
Ephb4 Control
Ephb4 CKO
50kDa
GAPDH
37kDa

Figure 8A

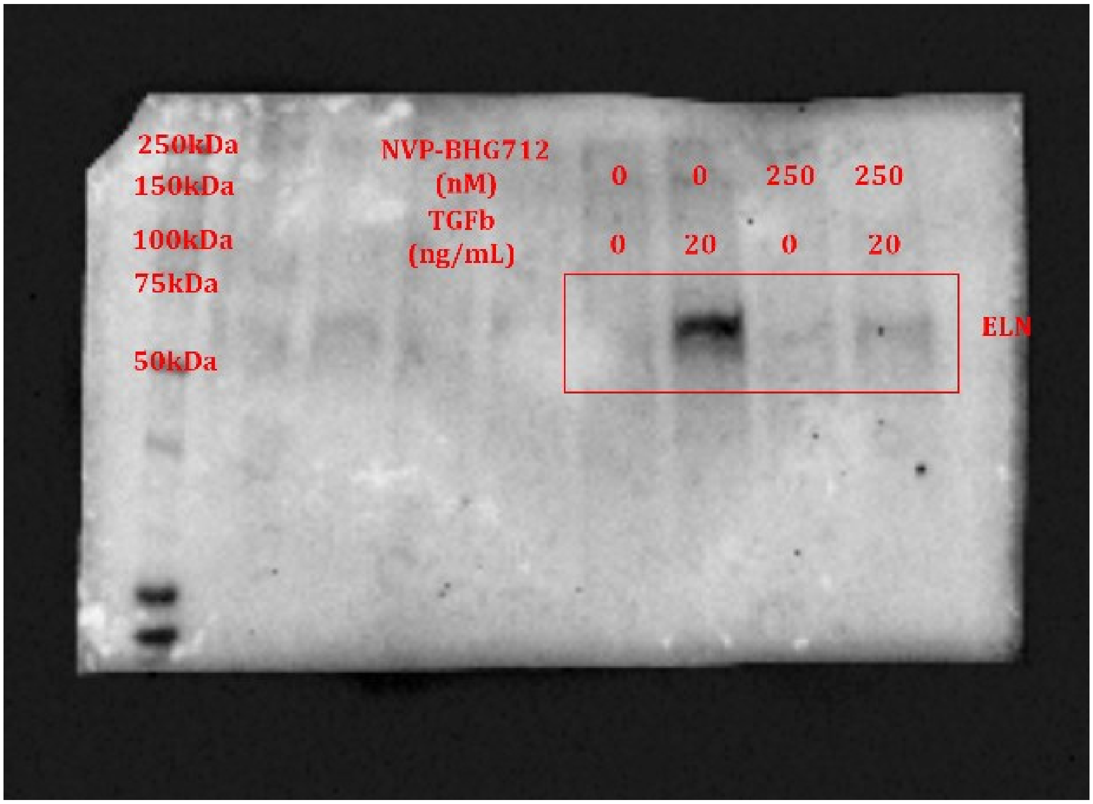
250kDa
150kDa
100kDa
75kDa
50kDa
NVP-BHG712 (nM)
0 0 250 250
TGFb (ng/mL)
0 20 0 20
ELN

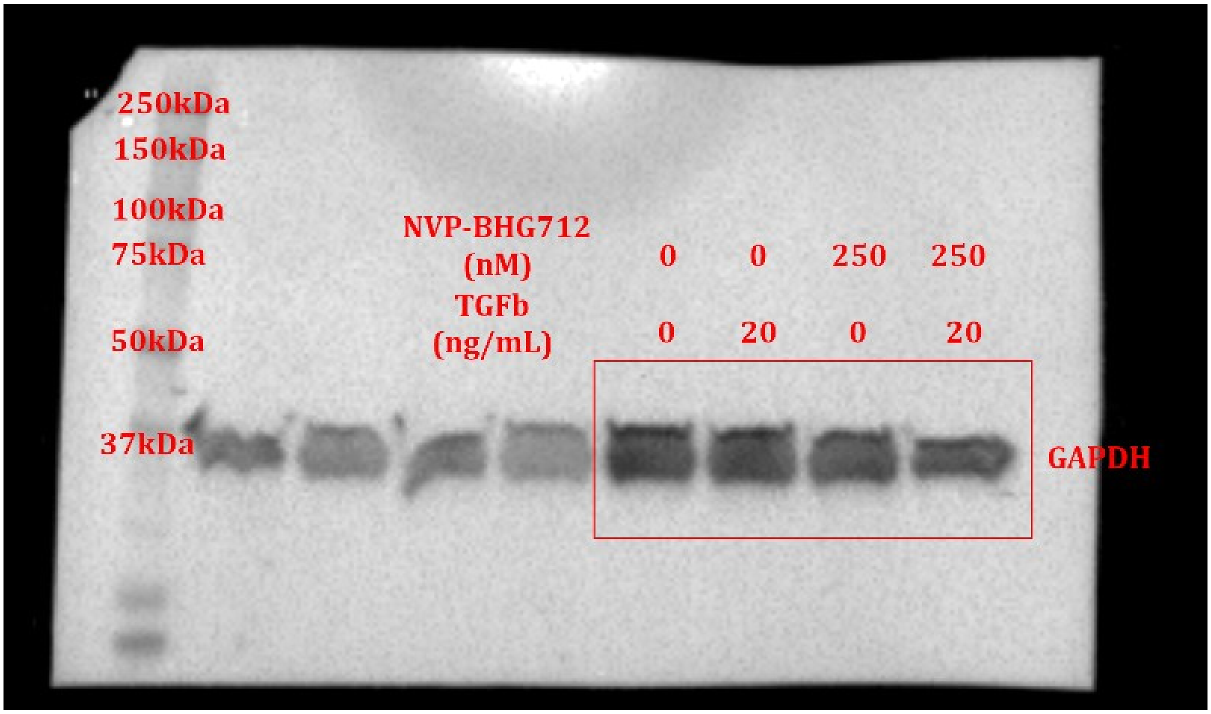
250kDa
150kDa
100kDa
75kDa
50kDa
37kDa
NVP-BHG712 (nM)
0 0 250 250
TGFb (ng/mL)
0 20 0 20
GAPDH

Figure 8B

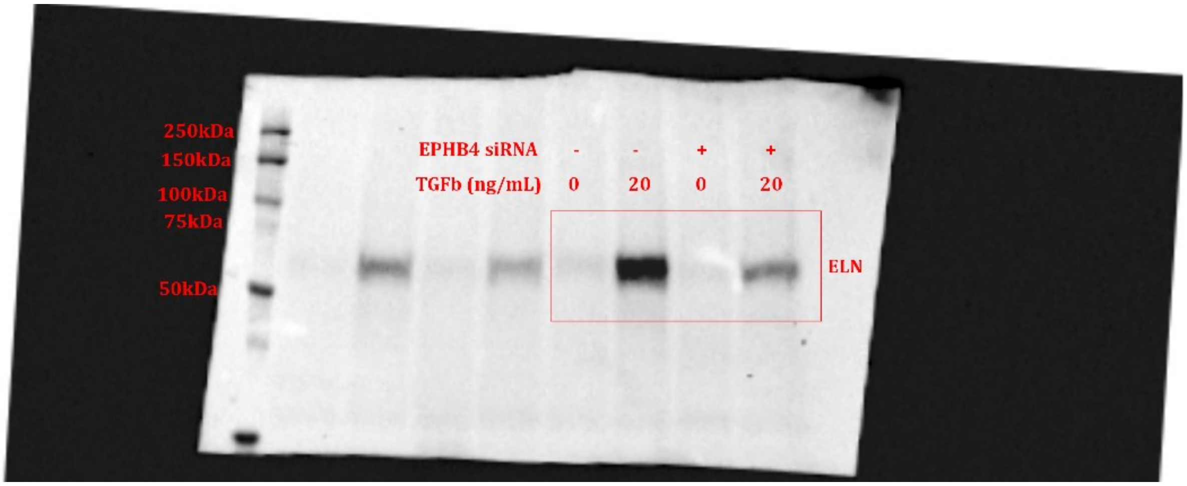
250kDa
150kDa
100kDa
75kDa
50kDa
EPHB4 siRNA - - + +
TGFb (ng/mL) 0 20 0 20
ELN

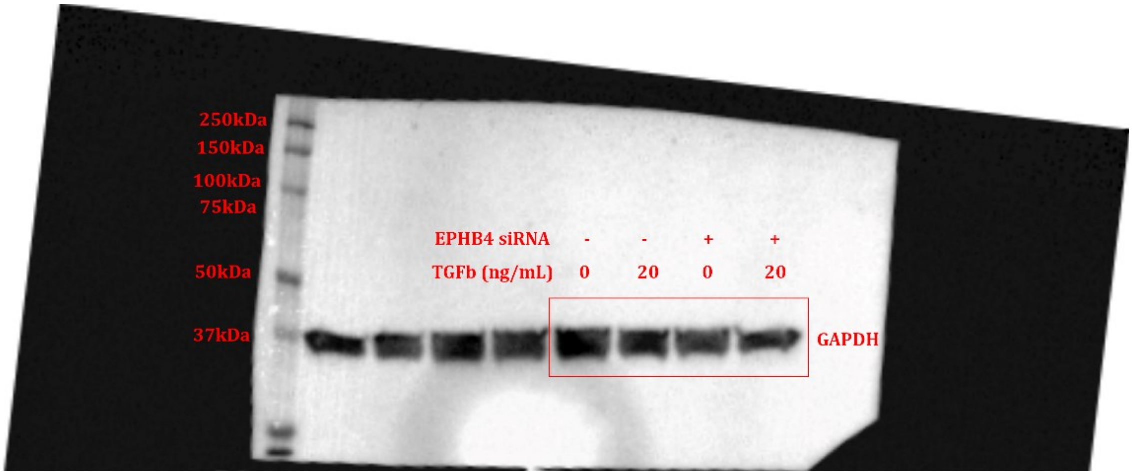
250kDa
150kDa
100kDa
75kDa
50kDa
37kDa
EPHB4 siRNA - - + +
TGFb (ng/mL) 0 20 0 20
GAPDH

Figure 8C

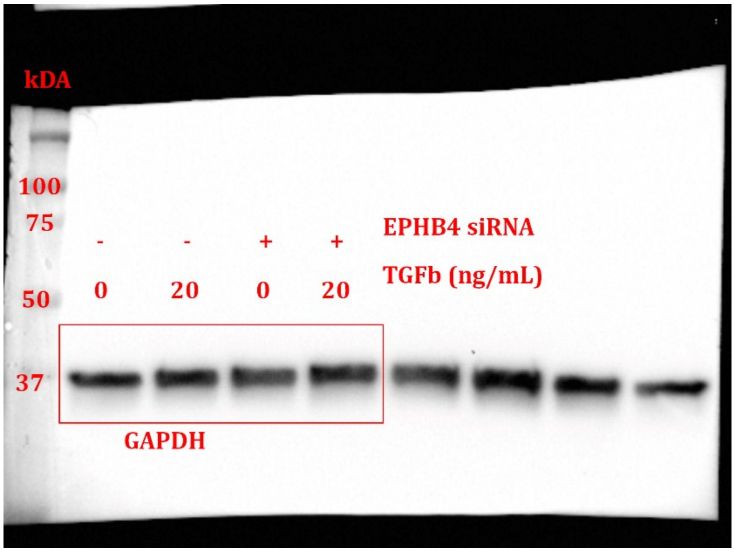
kDA
100
75
50
37
EPHB4 siRNA
- - + +
TGFb (ng/mL)
0 20 0 20
GAPDH

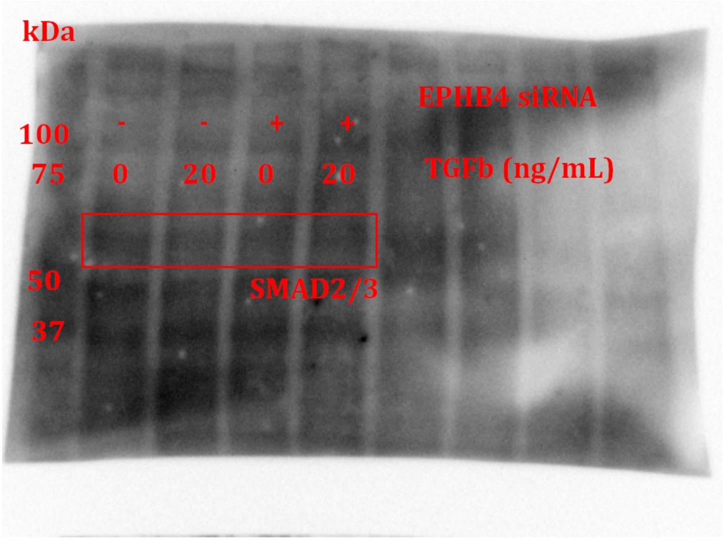
kDa
100
75
50
37
EPHB4 siRNA
- - + +
TGFb (ng/mL)
0 20 0 20
SMAD2/3

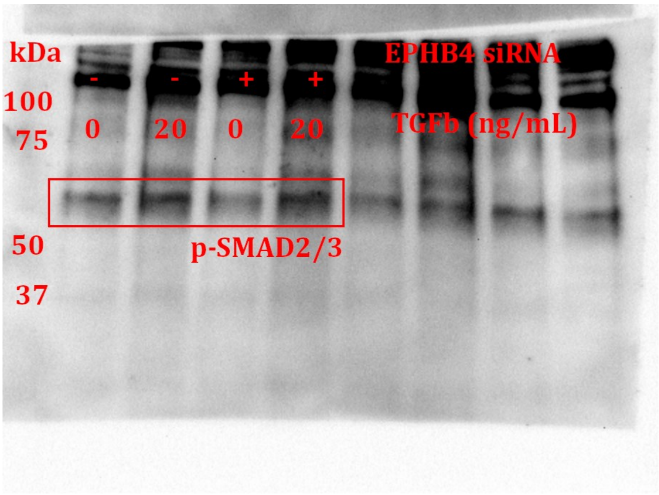
kDa
100
75
50
37
EPHB4 siRNA
- - + +
TGFb (ng/mL)
0 20 0 20
p-SMAD2/3

Supplementary Figure 2B

EphB4 (top left panel)

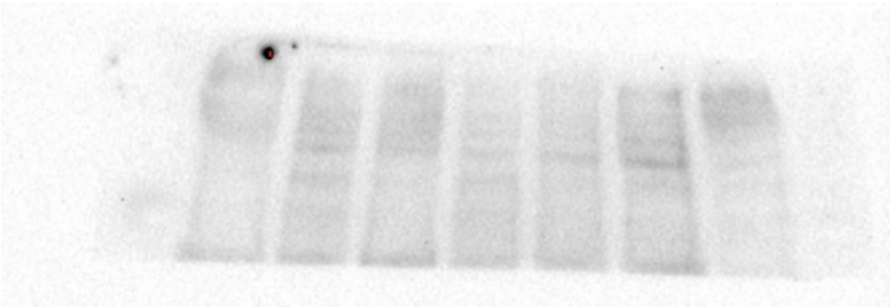

GAPDH (bottom left panel)

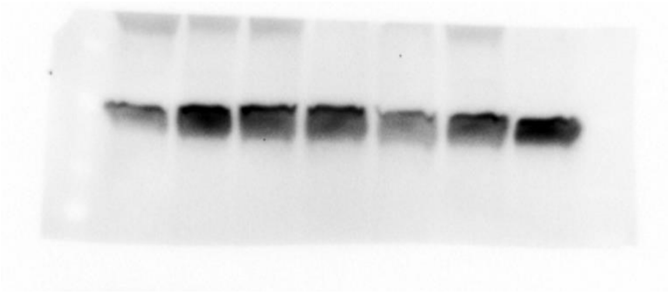

EphB4 (top right panel)

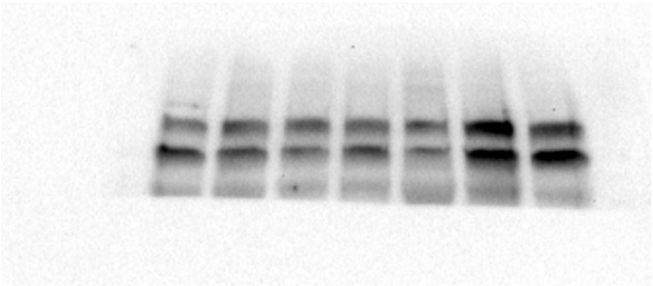

GAPDH (bottom right panel)

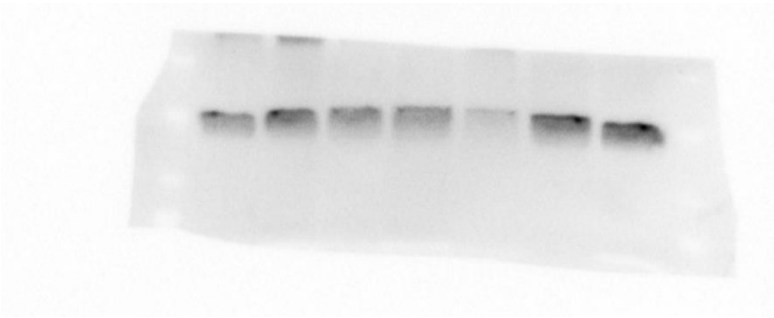

Supplementary Figure 4A

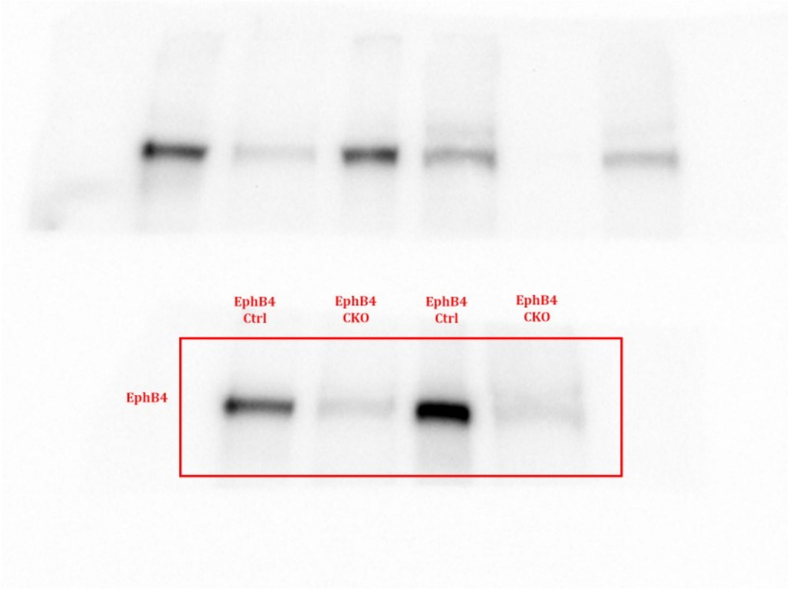

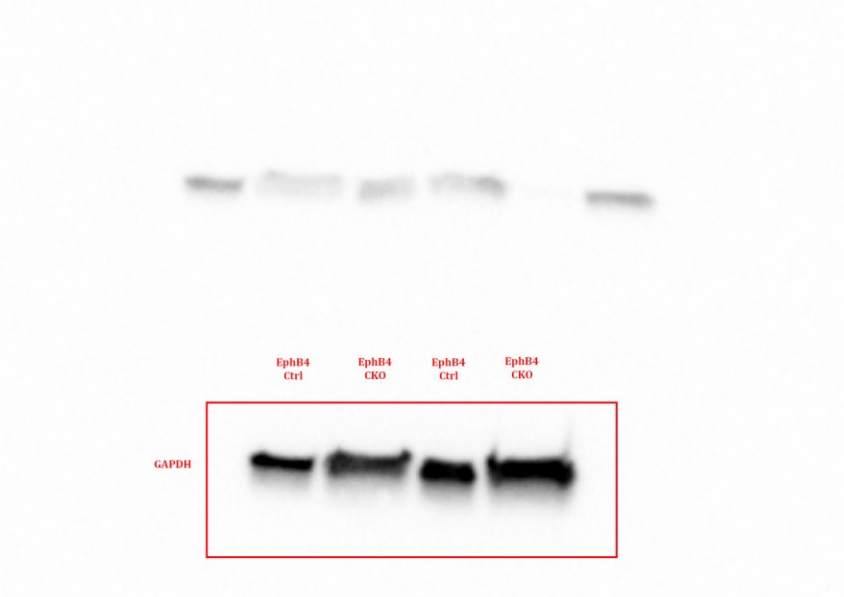

Supplementary Figure 5

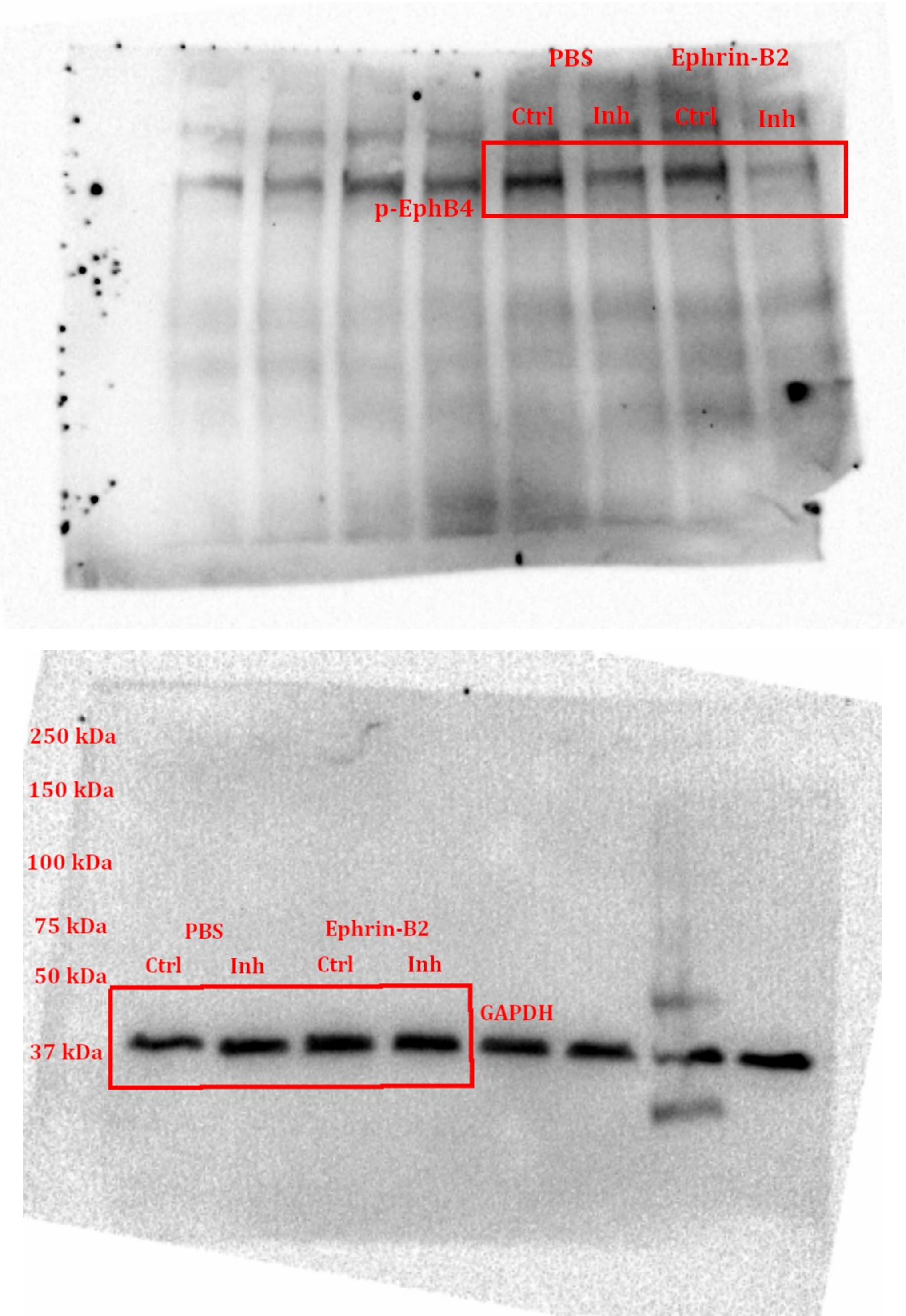

Supplement: Unedited blot and gel images [file jciinsight-10-189156-s126.pdf]
